# An approach to analyze spatiotemporal patterns of gene expression at single-cell resolution in *Candida albicans*-infected mouse tongues

**DOI:** 10.1128/msphere.00282-24

**Published:** 2024-08-22

**Authors:** Elena Lindemann-Perez, Diana L. Rodríguez, J. Christian Pérez

**Affiliations:** 1 Department of Microbiology and Molecular Genetics, McGovern Medical School, The University of Texas Health Science Center at Houston, Houston, Texas, USA; University of Michigan Michigan Medicine, Ann Arbor, Michigan, USA

**Keywords:** *Candida albicans*, hybridization chain reaction, RNA-FISH, *Candida*-host interactions, oropharyngeal candidiasis, *Candida*-infected tissue, microbial transcript visualization

## Abstract

Microbial gene expression measurements derived from infected organs are invaluable to understand pathogenesis. However, current methods are limited to “bulk” analyses that neglect microbial cell heterogeneity and the lesion's spatial architecture. Here, we report the use of hybridization chain reaction RNA fluorescence *in situ* hybridization (HCR RNA-FISH) to visualize and quantify *Candida albicans* transcripts at single-cell resolution in tongues of infected mice. The method is compatible with fixed-frozen and formalin-fixed paraffin-embedded tissues. We document cell-to-cell variation and intriguing spatiotemporal expression patterns for *C. albicans* mRNAs that encode products implicated in oral candidiasis. The approach provides a spatial dimension to gene expression analyses of host-*Candida* interactions.

**IMPORTANCE:**

*Candida albicans* is a fungal pathobiont inhabiting multiple mucosal surfaces of the human body. Immunosuppression, antibiotic-induced microbial dysbiosis, or implanted medical devices can impair mucosal integrity enabling *C. albicans* to overgrow and disseminate, causing either mucosal diseases such as oropharyngeal candidiasis or life-threatening systemic infections. Profiling fungal genes that are expressed in the infected mucosa or in any other infected organ is paramount to understand pathogenesis. Ideally, these transcript profiling measurements should reveal the expression of any gene at the single-cell level. The resolution typically achieved with current approaches, however, limits most gene expression measurements to cell population averages. The approach described in this report provides a means to dissect fungal gene expression in infected tissues at single-cell resolution.

## INTRODUCTION

Microbial gene expression measurements (e.g., RT-qPCR, RNA-Seq) collected directly from infected organs are essential to study pathogenesis. Infection-specific functions, for example, have been discovered using dual RNA-Seq of *Yersinia pseudotuberculosis*-infected lymphatic tissues (1) or targeted RNA-Seq of *Candida albicans*-infected tongue epithelial sheets (2). Dual RNA-Seq data sets (for example, see references 3–6) have typically used *ex vivo* infected host cells rather than organs. Currently, most gene expression analyses of organs infected with bacterial or fungal pathogens rely on “bulk” approaches, i.e., measurements derived from homogenized tissue samples. The infection sites' spatial context and architecture are lost with these methods. Likewise, cell-to-cell variation in gene expression of both host and microbe cannot be recorded. Exploring these features of host-pathogen interactions requires methods capable of generating spatially resolved gene expression measurements at single-cell resolution.

The fungus *Candida albicans* is a ubiquitous pathobiont residing in the oral cavity and digestive tract of humans. Oral mycobiome analyses based on DNA sequencing have shown that most healthy adults carry this yeast as part of the mouth's normal microbiota (7–9). The fungus, nonetheless, can overgrow in a diverse group of individuals causing oral thrush (oropharyngeal candidiasis), a debilitating mucosal disease. Multiple fungal and host factors contribute to *C. albicans* oropharyngeal candidiasis (see references 10, 11 for recent reviews). On the fungus side, these include adhesins such as hyphal wall protein 1 (Hwp1) and agglutinin-like sequence (Als) proteins (12–15); candidalysin, a secreted fungal peptide toxin (16); regulators of biofilm formation (17); endocytosis factors (18); and metabolic adaptations (2), among others.

Although important strides have been made with single-cell RNA sequencing in fungi and bacteria (19–22), the accurate quantification of transcripts at single-cell resolution remains challenging for unicellular organisms. Current efforts in microbial single-cell transcriptomics are still largely focused on “pure” *in vitro* cultures due to diverse challenges that need to be addressed when dealing with infected tissues, including complex cell wall structures and low mRNA abundance (23). Although PETRI-Seq (24), Micro-SPLiT (22), and 10× Chromium technology (21) have made possible to measure cell-to-cell heterogeneity with single-cell RNA-Seq in yeast and bacteria, dissecting spatial patterns of gene expression is not possible with these technologies. Recent advances in spatial host–microbiome sequencing and metatranscriptomics (25, 26) are still limited to low spatial resolution (~55–100 µM). More specialized, spatially resolved approaches are clearly needed.

## RESULTS

### Motivation

**Fig 1:**
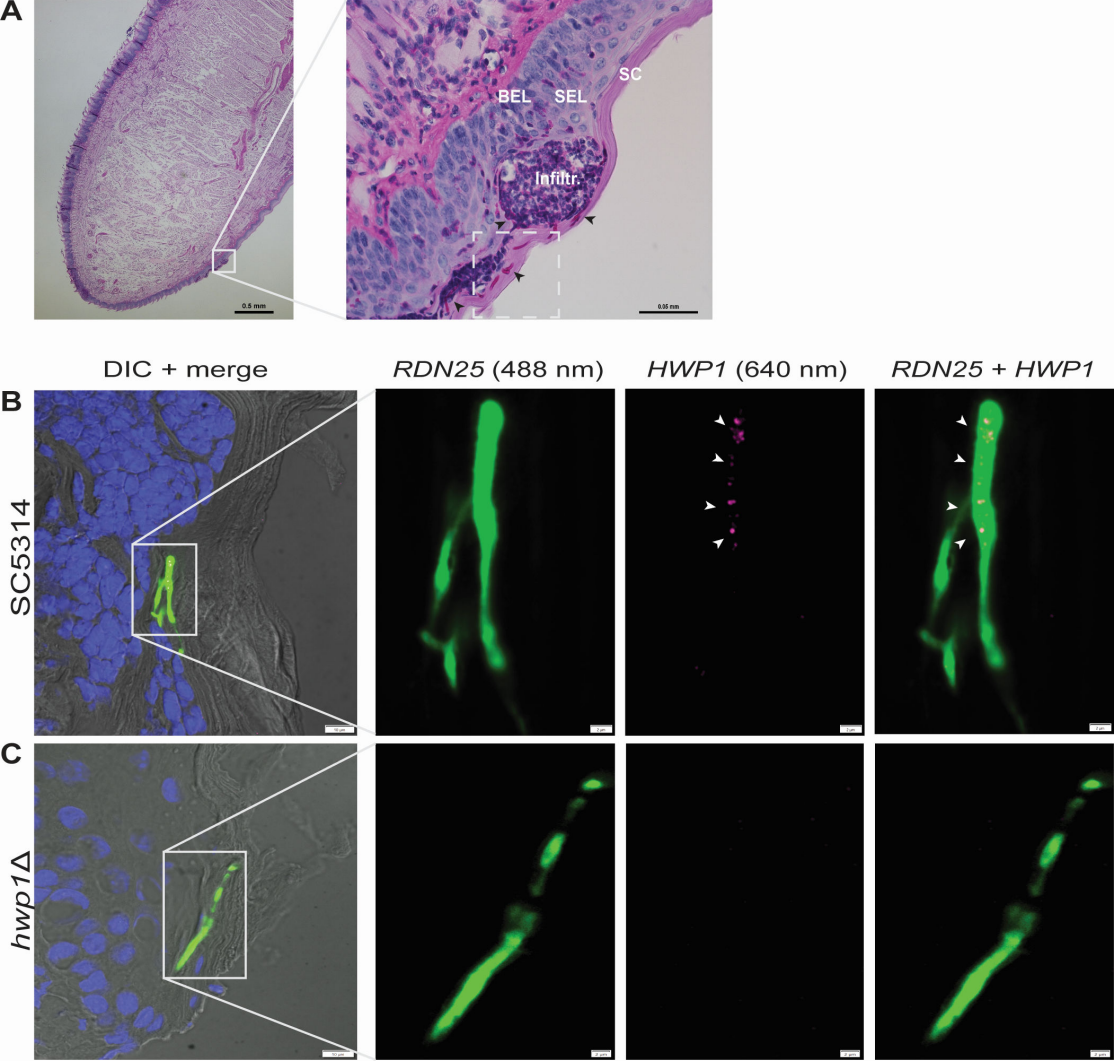
Fungal transcript visualization in *C. albicans*-infected mouse tongues. (**A**) PAS-stained sagittal section of murine tongue infected with *C. albicans*. Inset in left panel is enlarged to the right to show infection site structure. SC, stratum corneum; SEL, suprabasal epithelial layer; BEL, basal epithelial layer; infiltr, immune cell infiltrate (purple). Black arrowheads point to *C. albicans* cells. Dashed square depicts approximate area of HCR visualization and analysis. Scale bar in left panel, 500 µm; in right panel, 50 µm. (**B** and **C**) Fixed-frozen tongue sections collected 28 h after infection from mice inoculated with *C. albicans* SC5314 wild-type (**B**) or an isogenic *hwp1* deletion strain (**C**). *Candida* hyphae (green) are detected with HCR probes and amplifiers that hybridize fungal rRNA. *HWP1* transcripts (purple) are indicated with white arrowheads. Nuclei from epithelial and immune cells are stained with DAPI (blue). Insets in left panels are enlarged to the right. Scale bars in left panels, 10 µm; in the enlarged images, 2 µm. Numbers in parenthesis are the wavelengths of the laser lines used for imaging.

We and others have adopted immunocompetent mice to study host and fungal determinants of *C. albicans* oral infections (27, 28). Histology sections of *C. albicans*-infected tongues from these rodents consistently show fungal cells embedded in diverse tissue microenvironments (27, 29) (Fig. 1A). For example, *C. albicans* hyphae penetrate one or more layers of the oral epithelium, including the stratum corneum and the suprabasal epithelial layer; immune cell infiltrates surround some infection foci but not others. Moreover, local nutrient availability may differ between oral epithelial layers and can change during disease progression as the fungus assimilates host nutrients and the host responds to the fungus, producing dynamic microenvironments (30). Such spatial and temporal heterogeneity, in combination with the stochasticity of gene expression, may generate heterogeneity in *C. albicans* cell populations within host niches. Exploring this notion requires methods that add spatial resolution to fungal transcript measurements in infected tissues.

### Visualizing fungal transcripts in *C. albicans*-infected mouse tongues

Hybridization chain reaction RNA fluorescence *in situ* hybridization (HCR RNA-FISH, herein termed HCR) is a powerful method to visualize transcripts at single-cell resolution (principles of the technique are outlined in reference 31 and references therein). While originally developed to detect host mRNAs in animal tissues (32), the high level of specificity and background reduction achieved with the latest version of the technology (33) has made possible to extend it to fungal cells grown in planktonic cultures (31). Here, we sought to establish HCR to detect fungal transcripts in *C. albicans*-infected tissues. There are, however, inherent challenges in applying FISH-related approaches to this endeavor. Difficulties include the low number of fungal cells relative to host cells and the permeabilization of the complex fungal cell wall while preserving the overall structure of the tissue.

We removed tongues from mice infected with the *C. albicans* strain SC5314 and established appropriate tissue pretreatment and permeabilization steps to enable the visualization of fungal transcripts from either fresh-frozen or formalin-fixed paraffin embedded tongues. As proof-of-concept, we first targeted the *HWP1* transcript because it encodes an adhesin that mediates covalent attachment to buccal cells (15) and it has extensively been implicated in *C. albicans* oral infections. To detect fungal cells in the tongues, we chose to target the abundant 25S ribosomal RNA (*RDN25*; probes were designed to hybridize to fungal specific sequences of this rRNA). Simultaneously probing with *RDN25* and an anti-*Candida* antibody or calcofluor white demonstrated that, under our experimental conditions, *RDN25* probing captured 80–90% of *Candida* cells in the tissue (Fig. S1 and S2). As shown in Fig. 1B, the procedure that we implemented revealed multiple puncta in the *C. albicans* hyphae corresponding to the *HWP1* transcript. The fluorescence was specific to *HWP1* because no comparable signal was apparent in tongues infected with the isogenic *C. albicans hwp1* knockout strain (Fig. 1C).

**Fig 2:**
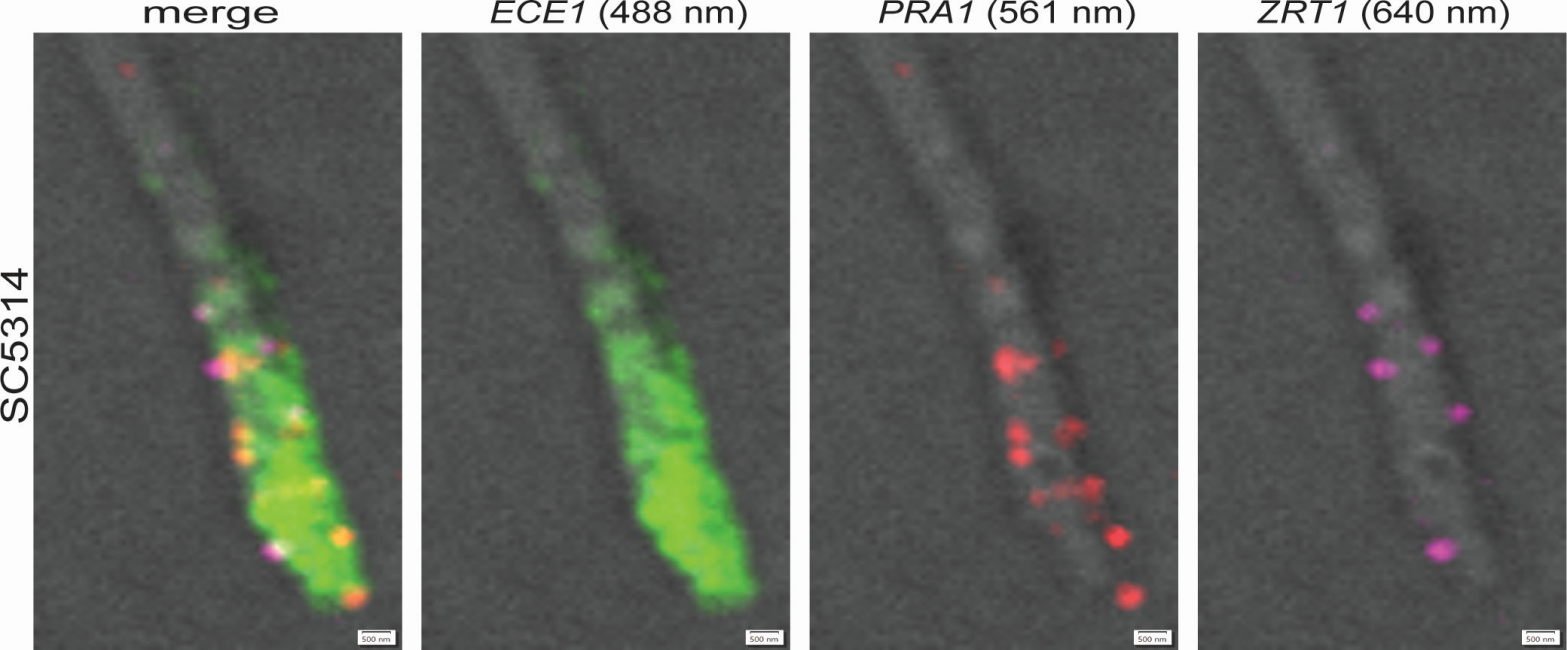
Localized expressions of *ECE1, PRA1*, and *ZRT1* in *C. albicans* hyphae. High-resolution images of multiplex HCR targeting *ECE1* (candidalysin), *PRA1*, and *ZRT1* transcripts in formalin-fixed paraffin-embedded sections. Tissue collected 48 h after infection. Scale bars, 0.5 µm. Numbers in parenthesis are the wavelengths of the laser lines used for imaging.

We next used HCR to visualize multiple fungal transcripts simultaneously (multiplex) while evaluating the method's performance with mRNAs of high, moderate, or low expression levels (based on publicly available RNA-Seq and Nanostring data sets [2, 34]). We chose the highly expressed *ECE1* gene that codes for candidalysin, a peptide toxin critical to cause damage in the oral epithelium (16), and two genes with moderate to low expression, *PRA1* and *ZRT1*, which encode a cell surface protein and a putative zinc transporter, respectively, that act together to sequester zinc from host tissues (35). As shown in Fig. 2, the fluorescent signal detected for each transcript reflected well their expected abundance. Remarkably, the *ECE1* transcript accumulated toward the hyphal tip (Fig. 2; Fig. S3) in every hypha that had *ECE1* fluorescent signal (~20% of hyphae were *ECE1*^+^). Furthermore, the *PRA1* and *ZRT1* transcripts localized primarily in the periphery of the hyphae. These findings are consistent with, and provide a mechanistic basis to, previous reports that have found the candidalysin peptide enriched in the hyphal tips (36) and the Pra1 protein accumulating in the cell periphery (37). The implication is that the mRNAs are the most likely molecules being mobilized to these locations for translation.

### Spatiotemporal features of *HWP1* expression during oral infection

**Fig 3:**
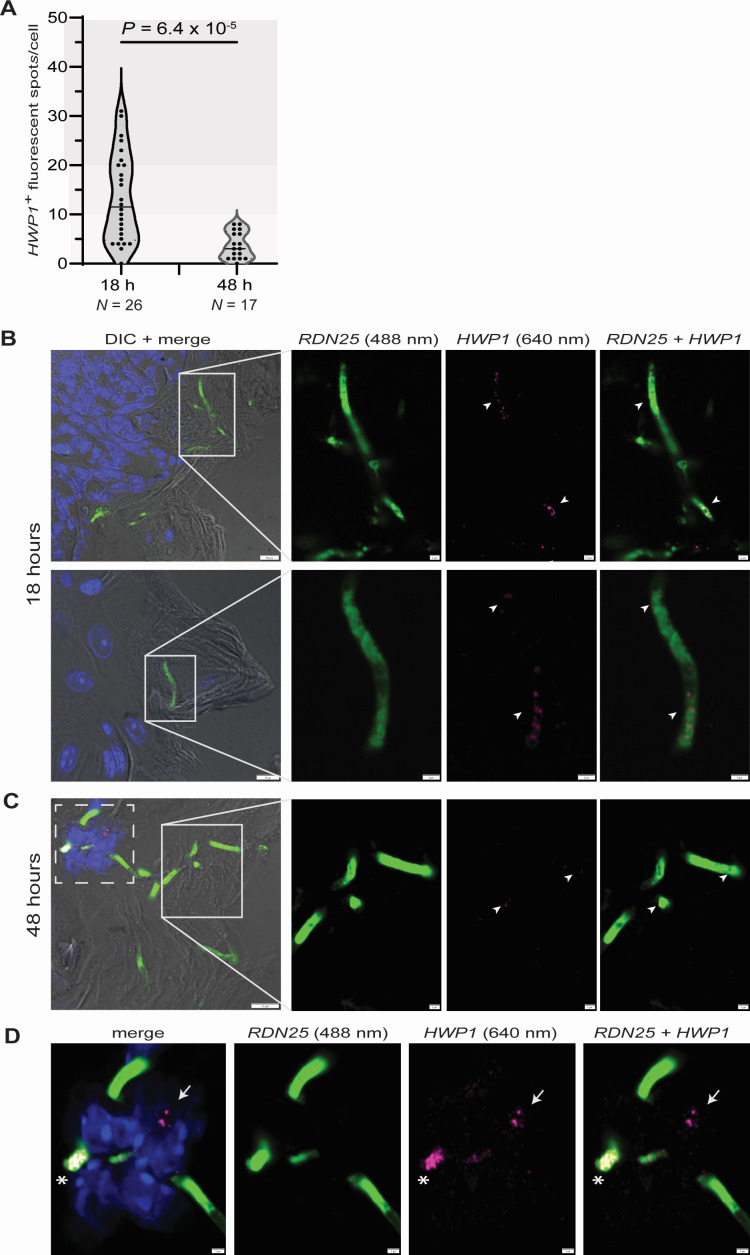
Quantification and distribution of *HWP1* transcript during infection. (**A**) *HWP1* transcript quantification in *C. albicans* hyphae located in the stratum corneum. Shown are violin plots where each dot corresponds to a single *C. albicans* hypha (length > 5 µm in MaxZ projection). Tongues were collected 18 or 48 h after infection. *N* is the number of hyphae quantified at each timepoint. Statistical analysis by the Mann–Whitney U-test. (**B** and **C**) Representative images showing *HWP1* transcript distribution and abundance in *C. albicans* hyphae laying in the tongue's stratum corneum. Formalin-fixed paraffin-embedded sections from tissues collected 18 h (**B**) or 48 h (**C**) after infection. *Candida* hyphae (green) are detected with HCR amplifiers and probes targeting fungal rRNA. *HWP1* transcripts (purple) are indicated with white arrowheads. Nuclei from epithelial and immune cells are stained with DAPI (blue). Insets in left panels are enlarged to the right. Dashed square in panel **C** is enlarged in panel **D**. Scale bars in left panels, 10 µm; in the enlarged images, 2 µm. (**D**) Aberrant patterns of *HWP1*'s fluorescent signal in *C. albicans* cells that had penetrated beyond the tongue's stratum corneum. White arrow points to *HWP1* signal that did not colocalize with fungal rRNA. Asterisk indicates tightly clustered foci of intense fluorescent signal. Nuclei from epithelial and/or immune cells are stained with DAPI (blue). Scale bars, 2 µm. Numbers in parenthesis are the wavelengths of the laser lines used for imaging.

*HWP1* expression is regulated by multiple transcription factors implicated in oral infections (17, 29). Therefore, we investigated in more detail the spatiotemporal patterns of expression of this key *C. albicans* adhesin during infection of the murine tongue. As shown in Fig. 3A, the number of *HWP1* transcripts varied widely from cell to cell (from 0 to 31 transcripts/cell; assuming that each fluorescent spot represents a single transcript molecule). For *C. albicans* hyphae located in the stratum corneum, we found that the amount of *HWP1* transcript per cell was higher at 18 h post inoculation compared to a later timepoint (48 h post inoculation) (Fig. 3A through C; Fig. S4 and S5), suggesting a more prominent role for this adhesin early in infection. Approximately 80–90% of hyphae found in the stratum corneum at either timepoint were *HWP1*^+^.

**Fig 4:**
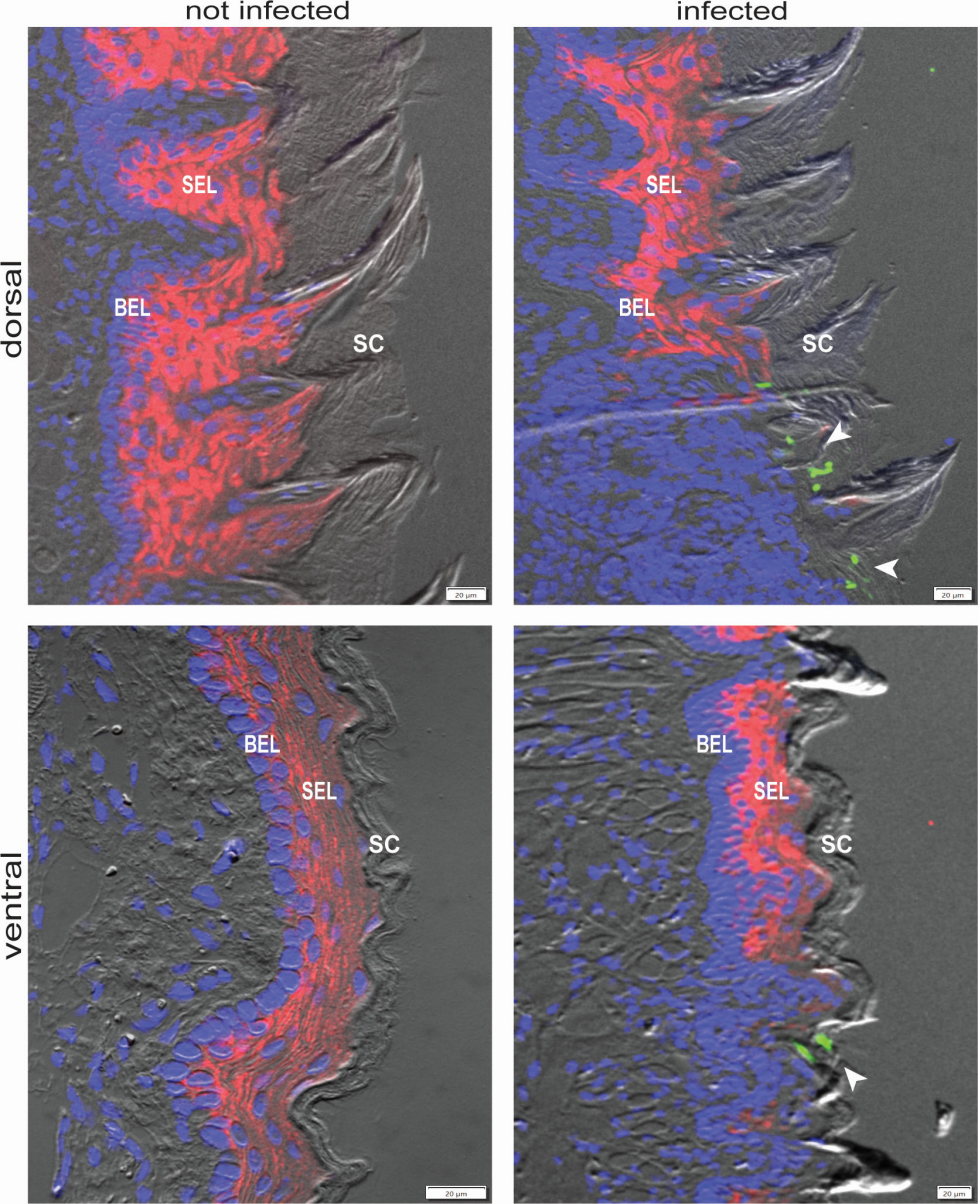
Visualizing host mRNA and *C. albicans* in infected tongues. Distribution of mouse keratin 13 mRNA (red) in dorsal and ventral tongue surfaces of control (not infected) and *C. albicans*-infected mice. Keratin 13 is a marker of the postmitotic suprabasal epithelial layer (SEL). Formalin-fixed paraffin-embedded sections were used. Mouse nuclei are stained with DAPI (blue). Notice keratin 13 signal abrogation in the area adjacent to *C. albicans* (green). BEL, proliferative basal epithelial layer; SC, stratum corneum. White arrowheads point to *C. albicans* cells, which are visualized with probes against *RDN25*. Scale bars, 20 µm.

When we inspected fungal cells that had penetrated deeper in the mucosa—and that often were adjacent to immune cell infiltrates (Fig. 1A)—the pattern and intensity of *HWP1*'s fluorescent signal became atypical (Fig. 3D; Fig. S5). In ~1/3 cases, *HWP1*'*s* fluorescent spots did not colocalize with fungal 25S ribosomal RNA. In other cells, the fluorescent signal accumulated in tightly clustered foci, which was in stark contrast to the uniform distribution of discrete puncta observed in *C. albicans* occupying the stratum corneum (Fig. 3B; Fig. S5). We speculate that fungal cell stress or damage resulting from the host's innate immune response may be partly responsible for the aberrant patterns observed in the more internal layer. Independently of the underlying cause, our single-cell transcript visualization points to notable differences among *C. albicans* cells depending on their location inside the oral mucosa.

### Visualizing fungal and host transcripts simultaneously

Finally, we assessed the feasibility of applying HCR to simultaneously visualize both fungal and mouse transcripts. On the host side, we targeted keratin 13 (K13), which is expressed in the subrabasal epithelial layer (SEL) of the tongue (Fig. 4), in agreement with previous reports that used antibodies (38). On the fungus side, we targeted *RDN25* (25S rRNA). We observed that the fluorescent signal corresponding to K13 extended uninterrupted throughout the SEL in uninfected mouse tongues, likely reflecting an intact SEL. In contrast to this pattern, *C. albicans*-infected tongues exhibited SEL portions with little or no K13 signal (Fig. 4). The disruption of K13 expression coincided with the presence of *C. albicans* cells adjacent to this area (Fig. 4). We speculate that the reduction in K13 signal corresponds to damaged SEL because superficial K13^+^ oral epithelial cells are the first to contact *C. albicans*. As part of the response to the fungus, the damaged SEL is sloughed and swallowed, in a process that helps to clear *C. albicans* (10, 38).

## DISCUSSION

In this report, we have adapted and expanded HCR to visualize fungal and host transcripts in tissue sections of *C. albicans*-infected murine tongues. We show that this method can successfully be applied to (i) detect and quantify at single-cell resolution fungal transcripts implicated in oral candidiasis, (ii) multiplex and visualize diverse mRNAs in the same *C. albicans* cell, (iii) image the distribution of specific transcripts along the *C. albicans* hyphae, (iv) determine whether certain genes are preferentially expressed by fungal cells located in defined layers of the infected tissue (e.g., stratum corneum *vs*. suprabasal epithelial layer), and (v) analyze host and *C. albicans* RNAs simultaneously. The exquisite combination of signal amplification and background reduction achieved with HCR in tongue tissues indicates that the procedure could easily be tailored to study additional isolates or other pathogenic fungi in a variety of mammalian tissues.

The patterns of *HWP1* expression that we document here suggest that there are differences between *C. albicans* cells depending on their location in the oral epithelium. Fungal cells laying in the stratum corneum exhibited a uniform distribution of *HWP1* mRNA signal (Fig. 3B and C), which could be readily enumerated. The reduction in *HWP1* expression that we found at the later infection timepoint (Fig. 3A) agrees with the observations made with *C. albicans* isolate 101 (2), a strain that exclusively colonizes the stratum corneum of the oral epithelium and, therefore, is not easily cleared from the tongue of immunocompetent mice (2, 39). In contrast to isolate 101, the standard reference strain SC5314 invades deeper in the tongue epithelium inducing an acute inflammatory response. It is plausible that stress or damage resulting from the host's innate immune response is partly responsible for the aberrant *HWP1* signal patterns (Fig. 3D) observed in *C. albicans* cells that penetrate deeper in the tissue. “Bulk” transcript measurements, which typically require tissue homogenization, would have missed these expression differences.

Limitations of the approach described here include the following. How many transcripts can be detected simultaneously in a multiplex assay depends on the number of excitation lasers and emission filters available in a microscope. Ten orthogonal amplifiers with diverse fluorophore labeling options are commercially available at the moment, which means that up to 10 different transcripts of interest can be visualized simultaneously. Stripping off and re-probing sections is, to the best of knowledge, not possible with this method. Differentiating *C. albicans* from the host tissue in these samples without any form of marker is cumbersome. While fluorescently tagged strains could serve for this purpose, the use of *RDN25* probes to “mark” *Candida* cells is the preferred strategy because it indicates successful cell wall permeabilization. This is critical to visualize the expression of other fungal transcripts of interest. Finally, absolute quantification of fluorescent puncta is only possible for transcripts that have relatively low abundance. Highly abundant transcripts like *RDN25* or that accumulate in a defined subcellular location like *ECE1* at the hyphal tips are challenging to enumerate.

Adding a spatial dimension to gene expression analyses of fungi-infected tissues opens new directions in fungal pathogenesis research. An area of significant interest is cell-to-cell variation in gene expression, which has been documented in populations of genetically identical fungal cells, yet its contribution to infection biology remains underexplored. Antifungal drug tolerance, wherein a subpopulation of fungal cells can continue to grow and multiply in the presence of antifungals while other genetically identical cells cannot, may be partly explained by cell-to-cell variation in gene expression (19). HCR can enable studies on the rise of antifungal drug tolerance in the host, i.e., during fungal infections. Likewise, this method may facilitate research on fungal cell specialization in biofilms (40, 41). Dual (host and fungus) HCR could also be used to interrogate host-fungus co-expression profiles generated by dual RNA-Seq, providing spatial resolution to the study of gene products mediating interactions between fungal and host cells.

## MATERIALS AND METHODS

### *C. albicans* strains and growth conditions

*C. albicans* clinical isolate SC5314 was used. The *hwp1* knockout has been previously described (29). *Candida* strains were routinely propagated in YPD (1% yeast extract, 2% peptone, and 2% dextrose) medium at 30°C.

### Mouse oral infections

Murine oral infections were conducted as previously described (27). Six- to 9-week-old, immunocompetent, female C57BL/6J mice (Jackson Laboratory) were used.

### HCR split-initiator probes and amplifiers

Target DNA sequences for *C. albicans* genes *RDN25* (*CR_08810W*), *HWP1* (*C4_03570W*)*, ECE1* (*C4_03470C*)*, PRA1* (*C4_06980W*), and *ZRT1* (*C4_06970C)* were obtained from the *Candida* Genome Database (http://www.candidagenome.org/). To visualize the suprabasal oral epithelial layer (SEL), we targeted the *Mus musculus* keratin 13 (*MmKRT13*; NM_010662.2) transcript. Split-initiator probe design followed criteria described before (31). Probe sets are listed in Table S1. HCR amplifiers with fluorophores B1-Alexa Fluor-546, B2-Alexa Fluor-647, and B3-Alexa Fluor-488 were obtained from Molecular Instruments, Inc.

### Tongue removal, fixation, and sectioning

The procedures outlined here and below can be found in a step-by-step protocol format in Supplemental Text. *C. albicans*-infected murine tongues were removed 18, 28, or 48 h post infection, halved longitudinally, and fixed overnight at 4°C in 10% buffered formalin. One half of the tongue was embedded in paraffin whereas the other half was frozen in an isopentane bath. For formalin-fixed paraffin-embedded (FFPE) samples, UTHealth's histology core facility carried out tissue processing, paraffin embedding, sectioning (5–6 μm), and mounting on baked Superfrost Plus slides (Fisher Scientific) following standard operating procedures. For fixed-frozen (FF) samples, formalin-fixed tongues were immersed in PBS solutions containing increasing amounts of sucrose (10%, 20%, and 30% sucrose in 1× PBS) at 4°C and embedded in Optimal Cutting Temperature (OCT) compound (Fisher Healthcare) using cryo-molds (VWR). The molds were placed in an isopentane bath until frozen and stored at −80°C. Cryostat sections (8 µm) were mounted on baked Gold ColorFrost Plus slides (Fisher Scientific), air dried for 2 h at −20°C, and stored at −80°C.

### Tissue pretreatment and permeabilization

FFPE samples were deparaffinized by baking at 65°C for 30 min (HybEZ II oven, ACD), rehydrated by immersing twice in 100% xylene and three times in 100% ethanol (each step for 4 min). FF samples were washed in 1× PBS for 5 min to remove the OCT compound, baked for 30 min, and post-fixed in 4% paraformaldehyde for 15 min at 4°C. The FF slides were subsequently immersed in increasing ethanol solutions (50%, 70%, and 100%) for 5 min each. Hydrogen peroxide solution (ACDBio) was applied to the rehydrated FFPE and FF sections, incubated for 10 min at room temperature, and washed afterwards in RNase-free water for 2 min.

To facilitate access to target RNA, we boiled slides for 10 (FFPE) or 5 (FF) min on a hot plate using 1× Target Retrieval Reagent (ACDBio). Subsequently, slides were washed in RNase-free water for 2 min, in 100% ethanol for 3 min, and dried for 5 min at 65°C. To digest the fungal cell wall, we treated sections with 40 µg/mL zymolyase 100T in 100 mM potassium phosphate buffer (pH 7.0) containing 1.2 M sorbitol and 30 mM β-mercaptoethanol for 25 min at 38°C in a humidified chamber. After washing with RNase-free water, 4.5 mg/mL proteinase K (Roche) or protease III (ACDBio) were added to FFPE or FF slides, respectively, and incubated for 5 min at 38°C (FFPE) or 30 min at 40°C (FF) in a humidified chamber.

### Probe hybridization and HCR

Sections were covered with 250 µL of probe hybridization buffer (30% formamide, 5× SSC, 9 mM citric acid [pH 6.0], 0.1% Tween 20, 50 µg/mL heparin, 1× Denhardt's solution, and 10% dextran sulfate) for 70–80 min at 39°C in a humidified chamber. The solution was removed and 75 µL of prewarmed probe solution containing the split-initiator probes (0.6–4 µL of 1 µM probe stock, depending on the abundance of the mRNA) in probe hybridization buffer was added. Slides were incubated in the humidified chamber for 18–22 h at 39°C. To remove excess probe, we washed slides in a (prewarmed) serial dilution (75%, 50%, 25%, and 0%) of probe wash buffer (30% formamide, 5× SSC, 9 mM citric acid [pH 6.0], 0.1% Tween 20, and 50 µg/mL heparin) in 5× SSCT (5× SSC and 0.1% Tween 20) for 15 min each at 37°C. After equilibration in 100% 5× SSCT for 5 min at room temperature, 800 µL of amplification buffer (5× SSC, 0.1% Tween 20, and 10% dextran sulfate) was added on top of the tissue sample, and the slides were incubated in a humidified chamber for at least 60 min at room temperature. In parallel, each fluorescently labeled hairpin (1.5 µL of 3 µM hairpin H1 and H2 stocks) was denatured separately at 95°C for 90 seconds, snap-cooled for 5 min on ice, and kept at room temperature in the dark for 30 min prior to dilution in 75 µL of fresh amplification buffer (per slide). After buffer removal, 75 µL of the hairpin solution was added on top of the tissue sample, and the slides were incubated at room temperature for 20–22 h in a humidified chamber. Slides were washed three times in 5× SSCT for 10 min protected from light. Slides were counterstained with DAPI and mounted with ProLong Diamond Antifade Mountant.

### Immunostaining

After removal of excess HCR hairpins (amplifiers) by rinsing the slides in 5× SSCT for 10 min, slides were incubated in blocking buffer (1× PBS DEPC, pH 7.4, and 5% FBS) for 30 min at room temperature and protected from light. An anti-*Candida* antibody (Cat. No. PA173154; ThermoFisher Scientific) coupled to fluorescein isothiocyanate (FITC) (1:500 dilution in 1× PBS DEPC, pH 7.4) was applied to the tongue sections and the slides were incubated overnight at 4°C in a humidified chamber. Sections were counterstained with DAPI in 5× SSCT and mounted with ProLong Diamond Antifade Mountant.

### Calcofluor white staining

After removal of excess HCR hairpins (amplifiers) by rinsing the slides in 5× SSCT for 10 min, slides were incubated in 0.1% (vol/vol) Fluorescent Brightener 28 disodium salt solution (Cat. No. 910090; Sigma-Aldrich) in 5× SSCT for 15 min at room temperature and protected from light. Slides were washed in 5× SSCT for 10 min and mounted with ProLong Diamond Antifade Mountant.

### Confocal microscopy

An Olympus IX-83 spinning-disk confocal microscope was used for image acquisition with cellSens software (v.2.3). Specifications used are as follows: excitation lasers, 405, 488, 561, and 640 nm; differential interference contrast (DIC) optics with 10×, 20×, 40×, 60×, and 100× objectives; piezo Z stage; wide field Xenon light source.

For *HWP1* transcript quantification (number of fluorescent puncta per *C*. *albicans* hypha), z-stacks of 0.25 nm were taken over the thickness of the section and aligned to maximal Z projections using cellSens software. *C. albicans* hyphae included in the quantification met the following criteria: location in the tongue's stratum corneum; length > 5 µm; exhibit even *RDN25* fluorescent signal. The enumeration of puncta per hyphae was done manually (unblinded).

### Statistical analysis

Differences in transcript distribution were evaluated by the Mann–Whitney *U*-test. Sections from ≥3 mice were included in every analysis.

## SUPPLEMENTAL MATERIAL

Supplemental figures msphere.00282-24-s0001.pdf Fig. S1 to S5.

Supplemental Text msphere.00282-24-s0002.pdf Step-by-step HCR protocol.

Table S1 msphere.00282-24-s0003.pdf Oligonucleotides used in the study.

ASM does not own the copyrights to Supplemental Material that may be linked to, or accessed through, an article. The authors have granted ASM a non-exclusive, world-wide license to publish the Supplemental Material files. Please contact the corresponding author directly for reuse.
