# Supplementary material for: An approach to analyze spatiotemporal patterns of gene expression at single-cell resolution in Candida albicans-infected mouse tongues
Source: mSphere. 2024 Aug 22;9(9):e00282-24. doi: 10.1128/msphere.00282-24 (PMC11423565; doi:10.1128/msphere.00282-24)
Supplement: Supplemental figures — Fig. S1 to S5. [file msphere.00282-24-s0001.pdf]

Supplemental Figure Legends

Figure S1. *C. albicans* detection using *RDN25* probes and FITC-labeled anti-*Candida* antibody. Formalin-fixed paraffin-embedded sections from tongues collected 18 h after infection with *C. albicans* were hybridized with HCR probes and amplifiers to detect fungal rRNA (*RDN25*, purple) and subsequently incubated with a FITC-labeled anti-*Candida* antibody (green). Nuclei from epithelial and immune cells are stained with DAPI (blue). Numbers in parenthesis at the top are the wavelengths of the laser lines used for imaging. Arrows point to selected hyphae (>5 µm in length) detected with the antibody but not with *RDN25*. Shown are 3 representative images (**A**-**C**). Scale bars, 20 µm.

Figure S2. *C. albicans* detection using *RDN25* probes and Calcofluor White. Formalin-fixed paraffin-embedded sections from tongues collected 18 h after infection with *C. albicans* were hybridized with HCR probes and amplifiers to detect fungal rRNA (*RDN25*, purple) and subsequently incubated with Calcofluor White stain (blue). Numbers in parenthesis at the top are the wavelengths of the laser lines used for imaging. Arrows point to hyphae (>5 µm in length) detected with Calcofluor White but not with *RDN25*. Shown are 3 representative images (**A**-**C**). Scale bars, 20 µm.

Figure S3. Localized accumulation of *ECE1* transcript at hyphal tips. Formalin-fixed paraffin-embedded sections from tongues collected 48 h (**A**) or 28 h (**B**-**E**) post infection were hybridized with HCR probes and amplifiers to detect *ECE1* transcript (green). Sections shown in (**C**-**E**) were hybridized with HCR probes and amplifiers to detect both *ECE1* (green) and *RDN25* (purple). Nuclei from epithelial cells are stained with DAPI (blue). Numbers in parenthesis at the top are the wavelengths of the laser lines used for imaging. Insets in left panels (**B**-**E**) are enlarged to the right. Scale bars, 2 µm in (**A**); 10 µm in (**B**); 5 µm in (**C**-**E**); and 2 µm in the enlarged images to the right.

Figure S4. *HWP1* transcript distribution 18 h post infection. Shown are 5 representative images (panels **A**-**E**) of *HWP1* transcript distribution in *C. albicans* hyphal cells laying in the tongue's stratum corneum 18 h post infection (formalin-fixed paraffin-embedded sections). Insets in the left panels are enlarged to the right. *Candida* hyphae (green) are detected with HCR probes and amplifiers targeting fungal rRNA (*RDN25*). *HWP1* transcripts are in purple and indicated with arrowheads. Nuclei from epithelial and immune cells are stained with DAPI (blue). Scale bars, 10 µm in DIC panels; 2 µm in enlarged images. Numbers in parenthesis at the top are the wavelengths of the laser lines used for imaging.

Figure S5. *HWP1* transcript distribution 48 h post infection. Representative images of *HWP1* transcript distribution in formalin-fixed paraffin-embedded tongue sections collected 48 h post infection. Panels (**A**-**C**) show hyphal cells laying in the tongue's stratum corneum. Panels (**D** and **E**) show aberrant patterns of *HWP1* signal in fungal cells that had penetrated beyond the stratum corneum. Insets in the left panels are enlarged to the right. *Candida* (green) detected with HCR probes and amplifiers targeting fungal rRNA (*RDN25*). *HWP1* transcripts are in purple and indicated with arrowheads. Asterisks indicate tightly clustered foci of intense fluorescent signal. White arrow points to *HWP1* signal that does not colocalize with *RDN25*. Nuclei from epithelial and immune cells are stained with DAPI (blue). Dashed squares in DIC panels of (**B**) and (**C**) are enlarged in panels (**D**) and (**E**), respectively. Scale bars, 10 µm in DIC panels; 2 µm in enlarged images. Numbers in parenthesis at the top are the wavelengths of the laser lines used for imaging.

Fig. S1

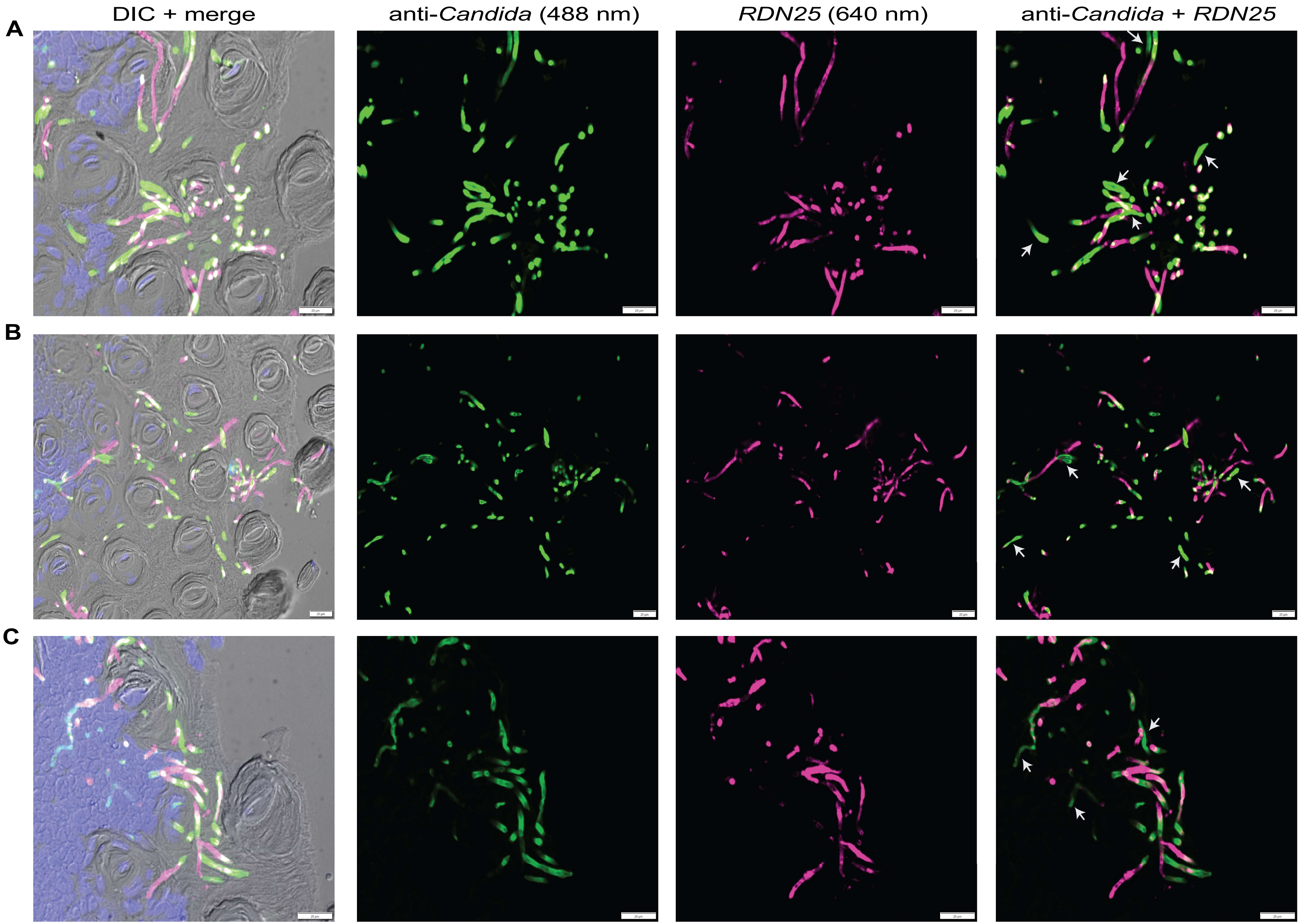
DIC + merge
anti-*Candida* (488 nm)
RDN25 (640 nm)
anti-*Candida* + *RDN25*
A
B
C

Fig. S2

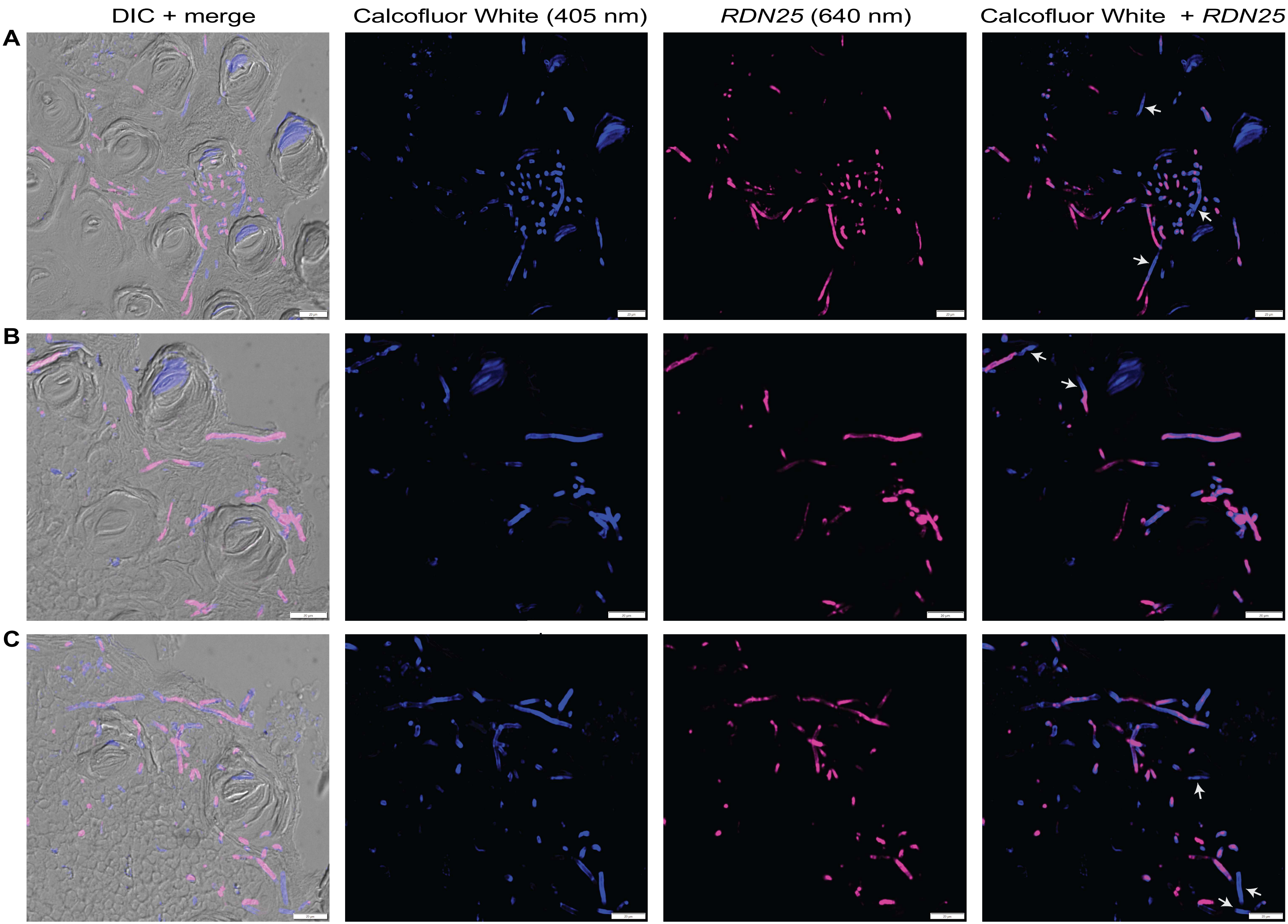
DIC + merge
Calcofluor White (405 nm)
RDN25 (640 nm)
Calcofluor White + *RDN25*
A
B
C

Fig. S3

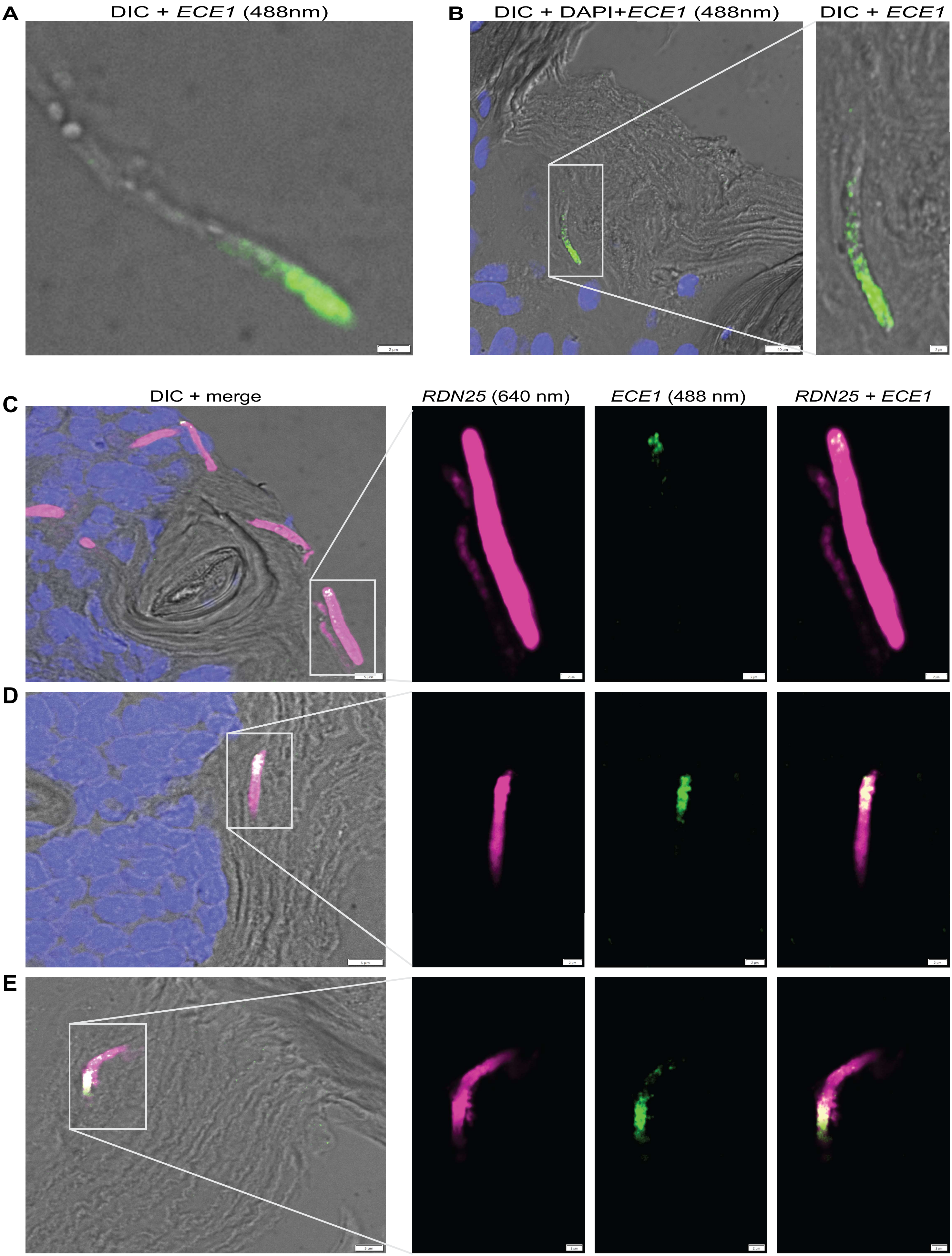

A
DIC + *ECE1* (488nm)
B
DIC + DAPI+*ECE1* (488nm)
DIC + *ECE1*
C
DIC + merge
RDN25 (640 nm)
ECE1 (488 nm)
RDN25 + *ECE1*
D
E

Fig. S4

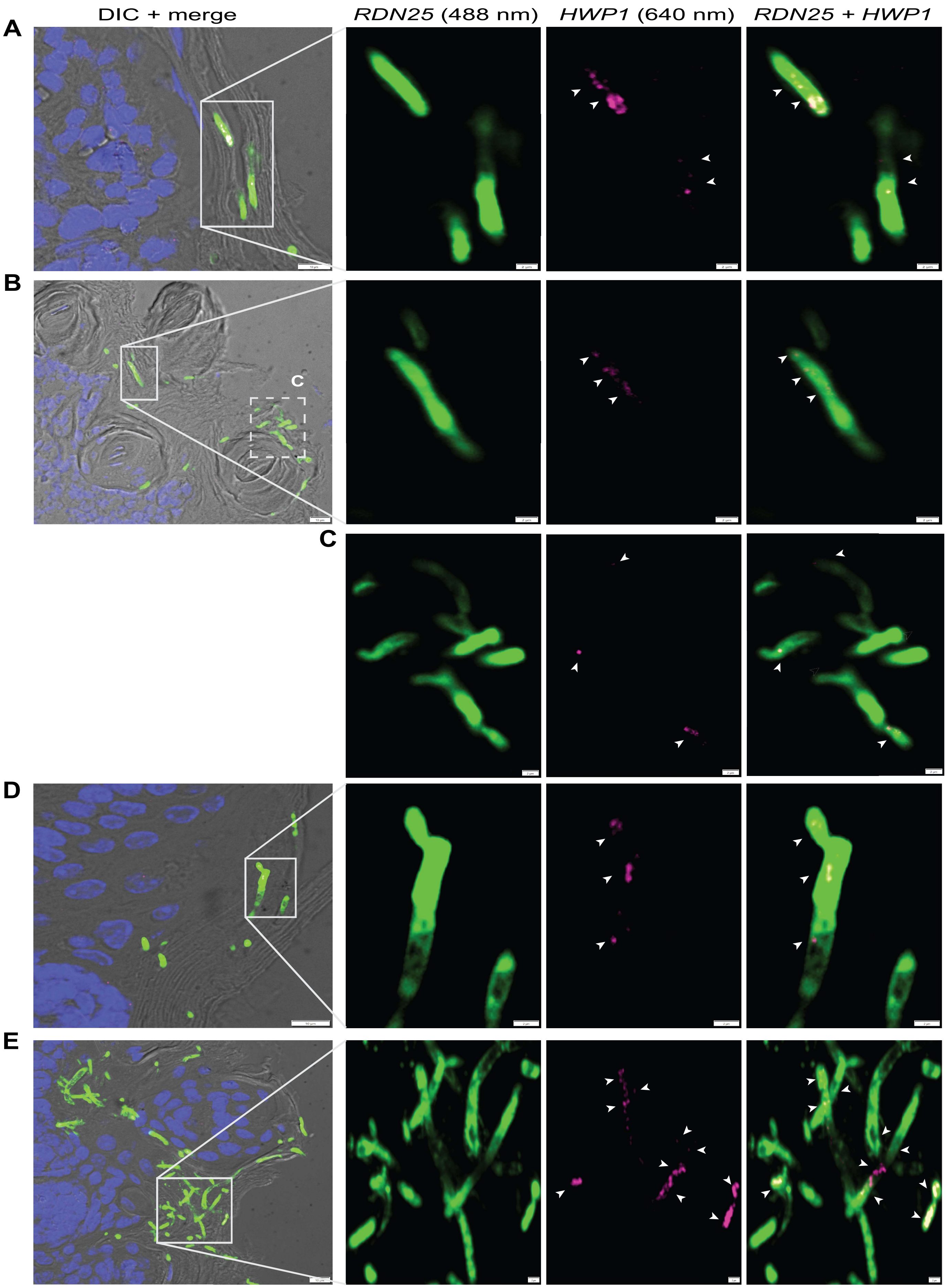

Fig. S5

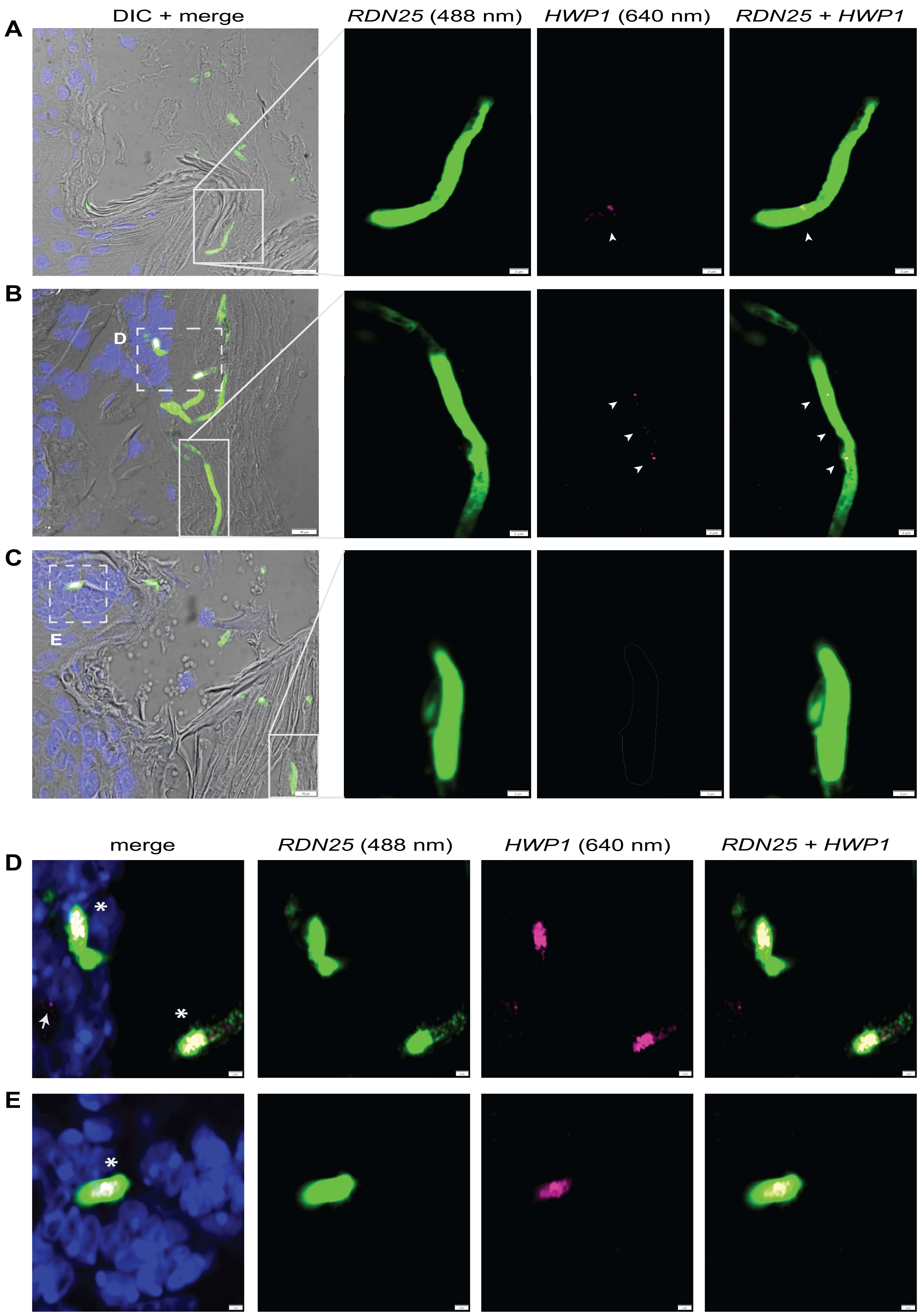

DIC + merge
RDN25 (488 nm)
HWP1 (640 nm)
RDN25 + HWP1
A
B
C
D
E
merge
RDN25 (488 nm)
HWP1 (640 nm)
RDN25 + HWP1
D
E
