# Supplementary material for: An approach to analyze spatiotemporal patterns of gene expression at single-cell resolution in Candida albicans-infected mouse tongues
Source: mSphere. 2024 Aug 22;9(9):e00282-24. doi: 10.1128/msphere.00282-24 (PMC11423565; doi:10.1128/msphere.00282-24)
Supplement: Supplemental Text — Step-by-step HCR protocol. [file msphere.00282-24-s0002.pdf]

Supplemental Text. Tissue processing and HCR step-by-step protocol

E. Lindemann-Perez, D.L. Rodríguez, and J.C. Perez. An approach to analyze spatiotemporal patterns of gene expression at single-cell resolution in *Candida albicans*-infected mouse tongues.

I. CHEMICALS AND ENZYMES

Description	Supplier	Cat. Number
RNaseZAP	SigmaAldrich	R2020
DEPC-treated water	Fisher Bioreagents	BP5611
Nuclease-free water	Ambion/Invitrogen	AM9930
Formamide (Deionized)	Ambion	AM9342
SSC Buffer, 20X concentrate	Sigma Aldrich	S6639
Citric Acid Anhydrous	Fisher Bioreagents	BP339
Denhardt's Solution (50X)	Invitrogen	750018
Tween 20	Sigma Aldrich	P9416
Heparin sodium salt from porcine intestinal mucosa	Sigma Aldrich	H3393
Dextran sulfate, MW>500,000	Sigma Aldrich	D8906
4% Paraformaldehyde in PBS	Alfa Aesar	J61899
D-Sorbitol	Sigma Aldrich	S6021
Sucrose	Sigma Aldrich	S0389
Xylenes	Sigma Aldrich	534056
Ethyl alcohol, pure, 200 proof	Sigma Aldrich	E7023
RNAScope H_2O_2 and Protease Reagent Kit	ACDBio	322381
RNAScope Target Retrieval Reagent Kit	ACDBio	322000
Proteinase K rec PCR grade	Roche	03115887001
Zymolyase 100T, 25mg	US Biological	Z1004
Phosphate-Buffered Saline (10x), pH 7.4	Invitrogen	AM9625
2-Mercaptoethanol, 99%	Sigma Aldrich	M3148
Protector RNase Inhibitor	Roche	03335399001
4',6'-diamidino-2-phenylindole (DAPI)	Invitrogen	D1306
ProLong Diamond Antifade Mountant	Invitrogen	P36961

II. GUIDELINES

- All glassware should be baked overnight at 180°C.
- Bench/Working space should be cleaned thoroughly with 70% EtOH and RNaseZAP.
- Work as RNase-free as possible during the duration of the experiment.

III. BUFFER RECIPES

Note: Probe hybridization buffer, probe wash buffer, and amplification buffer can also be purchased from Molecular Instruments.

Probe hybridization buffer (Prepare in 50 ml tube, make 10 ml aliquots, and store at **-20°C**)

Component	For 40 ml	Final concentration
formamide	12 ml	30%
20X sodium chloride sodium citrate (SSC)	10 ml	5x
1 M citric acid, pH 6	360 µl	9 mM
10% Tween 20	400 µl	0.1%
10 mg/ml heparin	200 µl	50 µg/ml
50x Denhardt's solution	800 µl	1x
50% dextran sulfate	8 ml	10%
	fill up to 40 ml with DEPC-treated water	

Probe wash buffer (Prepare in 0.5 L bottle, make 50 ml aliquots, and store at **-20°C**)

Component	For 400 ml	Final concentration
formamide	120 ml	30%
20X sodium chloride sodium citrate (SSC)	100 ml	5x
1 M citric acid, pH 6	3.6 ml	9 mM
10% Tween 20	4 ml	0.1%
10 mg/ml heparin	2 ml	50 µg/ml
	fill up (170.4 ml) with DEPC-treated water	

Amplification buffer (Prepare in 50 ml tube, make 10 ml aliquots, and store at **4°C**)

Component	For 40 ml	Final concentration
20X sodium chloride sodium citrate (SSC)	10 ml	5x
10% Tween 20	400 µl	0.1%
50% dextran sulfate	8 ml	10%
	fill up to 40 ml with DEPC-treated water	

5x SSCT (Prepare 1 L, store at **room temperature**)

Component	For 1 L	Final concentration
20X sodium chloride sodium citrate (SSC)	250 ml	5x
10% Tween 20	10 ml	0.1%
	fill up (740 ml) with DEPC-treated water	

50% Dextran sulfate (prepare in 50 ml tube, make 8 ml aliquots, and store at **-20°C**)

Component	For 40 ml	Final concentration
Dextran sulfate powder	20 g	50%
	fill up to 40 ml with Nuclease-free water (add water gradually)	

10 mg/ml heparin (prepare in 50 ml tube, make 10 ml aliquots, and store at **4°C**)

Component	For 50 ml	Final concentration
Heparin (store powder at RT)	500 mg<br>fill up to 50 ml with Nuclease-free water<br>→ filter-sterilize 0.2 µm	10 mg/ml

1M citric acid pH 6 (prepare in 50 ml tube and store at **room temperature**)

Component	For 50 ml	Final concentration
Citric acid	10.5 g	1 M
	fill up to 30 ml with Nuclease-free water<br>→ adjust pH to 6.0 with NaOH<br>→ filter-sterilize 0.2 µm	

Zymolyase buffer (prepare fresh just before use)

Component	For 10 ml	Final concentration
Potassium phosphate buffer, pH 7.0	1 ml	100 mM
3M Sorbitol	4 ml	1.2 M
β-mercaptoethanol	21 µl	30 mM
Zymolyase 100T, 25mg/ml	16 µl	40 µg/ml
RNase Inhibitor	5 µl	
	mix	

Proteinase K solution (prepare fresh just before use)

Add 1 µl Proteinase K to 3 ml of PBS-DEPC. Mix by inverting the tube.

5x SSCT-DAPI 1:1000 solution (prepare fresh before use and keep protected from light)

Add 40 µl DAPI (5mg/ml) to 40 ml 5x SSCT

Hairpins (amplifiers)

Purchased from Molecular Instruments:

B1 – Alexa Fluor 546, 600 pmol

B2 – Alexa Fluor 647, 600 pmol

B3 – Alexa Fluor 488, 600 pmol

→ Make 10-20 µl aliquots of each hairpin (H1 and H2) and store at **-20°C**.

Split-initiator probes

Probes were designed with Serial Cloner (v 2.6.1) according to parameters described before [Choi *et al*., 2018; Moreno-Velásquez & Pérez, 2021]:

(i) 25 nt target mRNA recognition sequence; *(ii)* T_m ranging from 60 to 85°C; and *(iii)* GC content between 40-75%. Each pair was validated by the CGD multi-genome NCBI BLAST+ tool to minimize potential off-target hybridization excluding probe pairs when both target sequences displayed >15-nt complementary to non-target mRNA.

Probe sets (Table S1) are ordered as standard, desalted oligonucleotides from SIGMA Millipore at 0.025 µmol scale.

→ Reconstitute oligos to 100 µM concentration with nuclease-free water (Ambion).

→ Mix all probes per target to 1 µM probe stock concentration.

IV. LABORATORY EQUIPMENT

Description	Supplier
Fume Hood	
HybEZ™ II Oven (110VAC)	ACDBio
Environmental chamber/37°C incubator	Thermo Scientific
Hotplate/Stirrer	VWR
PCR thermocycler	BioRad

V. OTHER MATERIALS

Description	Supplier	Cat. Number
SuperFrost Plus Slides	Fisher Scientific	12-550-15
Gold ColorFrost Plus Slides	Fisher Scientific	9951GLPLUS-006
Optimal Cutting Temperature (O.C.T.) compound	Tissue-Tek	4383
Cryomold standard	Tissue-Tek	4557
ImmEdge Hydrophobic Barrier Pen	Vector Laboratory	H-4000
Coplin jar (glass) or Tissue-Tek racks		
Microscope Slide Staining Rack	Electron Microscopy Sciences	71400
Kimwipes or other absorbent paper		
2 l heavy-duty glass beaker		
HybEZ Humidity Control Tray (with lid)	ACDBio	310012
EZ-Batch Slide Holder (Boekel)	ACDBio	321716
Blotting sheet	ACDBio	310025
HybriSlips Hybridization Coverslips	Electron Microscopy Sciences	70329-22
Micro Cover Glass #1.5, 22x40 mm	Electron Microscopy Sciences	72204-03
PCR tubes		

VI. PROCEDURES

1. Mouse tongue processing

1.1 Remove mouse tongue and cut it longitudinally in half.

1.2 Fix both tongue halves overnight at 4°C in 10% buffered formalin solution [Meir *et al*., 2018].

1.3 For Formalin-Fixed Paraffin-Embedded (FFPE) sections:

a. Transfer one tongue half to embedding cassette (Tissue-Tek Biopsy – Chamber Cassette).
Note: Up to three tongue halves can be placed in one cassette.

b. Tissue processing, paraffin embedding, sectioning (5-6 µm) and mounting on baked SuperFrost Plus slides is carried out by UTHealth's histology core facility following standard operating procedures.
Note: Do not mount more than one section per slide. Place sections in the center of the slide.

c. Dry slides overnight at 37°C.
d. Store the slides at room temperature (RT) with desiccant *(up to 3 months).*

1.4 For Fixed-Frozen (FF) sections:

a. Immerse formalin-fixed tongues in 10% sucrose - 1x PBS-DEPC solution (in 15 ml conical tube) at 4°C until the tissue sinks to the bottom of the tube.
b. Repeat this step with 20% sucrose - 1x PBS-DEPC at 4°C.
c. Repeat this step with 30% sucrose - 1x PBS-DEPC at 4°C.
d. Embed the tongue in OCT compound in cryo-molds.
Note: Up to three tongue halves can be placed in one cassette.
e. Place the cryo-mold in isopentane bath until frozen.
f. Store the molds in an airtight container at -80°C.
g. For preparation of sections, equilibrate the tissue block at -20°C for at least 30 min in a cryostat.
h. Cut 8 µm-thick sections.
i. Mount the section on baked Gold ColorFrost Plus slides.
Note: Do not mount more than one section per slide. Place sections in the center of the slide.
j. Air dry the slides for 2 h at -20°C.
k. Store the slides with desiccant at -80°C.
Note: Use sectioned tissue within three months.

2. Deparaffinization and rehydration of FFPE sections (go to step **3.** for Fixed-frozen slides)

2.1 Deparaffinize by baking the slides at 65°C for 30 min in HybEZ II oven.
2.2 Rehydrate the slides by immersing in 100% xylene (fume hood!) for 4 min in coplin jar at RT.
2.3 Repeat the step with 100 % xylene for 4 min at RT.
2.4 Transfer the slides to coplin jar with 100% EtOH at RT for 4 min.
2.5 Repeat the step **twice** with 100% EtOH for 4 min each at RT.
2.6 Bake the slides for 5 min in HybEZ II oven.

3. O.C.T. removal and rehydration of FF sections (skip for FFPE section)

3.1 Wash the slides in 1xPBS-DEPC for 5 min in a coplin jar to remove the O.C.T.
3.2 Bake the slides for 30 min in HybEZ oven.
3.3 Fix the sections by immersing the slides in cold 4% PFA for 15 min at 4°C (coplin jar).
3.4 Remove the slides from 4%PFA and immerse in 50% EtOH for 5 min at RT (coplin jar).
3.5 Remove the slides and place in 70% EtOH for 5 min at RT (coplin jar).
3.6 Remove the slides from 70% EtOH and immerse in 100% EtOH for 5 min at RT (coplin jar).
3.7 Remove the slides and place in new 100% EtOH for 5 min at RT (coplin jar).
3.8 Remove the slides from 100% and let them air dry for 5 min at RT.

4. Application of hydrogen peroxide solution to dehydrated FFPE or FF slides

4.1 Place the rehydrated slides on the bench and apply 5-8 drops of Hydrogen Peroxide solution (from RNAScope H_2O_2 and Protease kit).
4.2 Incubate the slides for 10 min at RT.
4.3 Remove H_2O_2 by flicking the slide on absorbent paper/kimwipes and immediately insert the slide into water (for RNA work) (coplin jar).
4.4 Wash the slides by moving up and down in the coplin jar 5 times.
4.5 Repeat step 4.4 with fresh water.
4.6 Place the slides in microscope slide staining rack.

5. Target retrieval (for FFPE and FF slides)

5.1 Dilute 10x Target Retrieval Reagent to 1x for a total volume of 400 ml in a 2 L heavy-duty glass beaker.
5.2 Place the beaker with the solution on the hotplate. Cover the beaker with foil and bring the solution to boil.
5.3 Once the 1x Target Retrieval Reagent reaches a mild boil (98-102°C), submerge the slide rack with samples into it. Check the temperature regularly with a thermometer and keep it between 98-102°C.
5.4 Boil **fixed-frozen mouse tongue samples for 5 min** and **FFPE samples for 10 min**.
5.5 Immediately transfer the hot slide rack into a separate staining dish containing in DEPC-treated water. Lift the rack 5x up and down.
5.6 Place the slides in 100% ethanol for 3 min at RT.
5.7 Dry the slides for 5 min at 65°C (HybEZ II oven).

6. Create a barrier around the section

6.1 Draw a barrier around the section with an ImmEdge hydrophobic barrier pen.
6.2 Let the slides dry completely for ca. 15-30 min (or OVERNIGHT) at RT.
Notes: i. Do NOT let the barrier touch the section.
ii. This can be an optional overnight stopping point.

7. Zymolyase treatment

7.1 Reduce the HybEZ II oven temperature to 38°C.
7.2 Place the slides in humidified chamber (HybEZ humidity control tray with wet blotting sheet).
7.3 Add 750 ul of freshly prepared Zymolyase-buffer on the section surface.
7.4 Incubate in the HybEZ II oven for 25 min at 38°C.
7.5 Remove the Zymolyase-buffer by flicking the slide on absorbent paper and immediately insert the slide into water (for RNA work) for 2 min.
7.6 Wash additional 2 min in fresh RNase-free water (coplin jar).
7.7 Remove liquid as much as possible with absorbent paper (Kimtech wipes).
7.8 Place the slides in humidified chamber.

8. Proteinase K treatment for FFPE slides (see section 9 for fixed-frozen slides)

8.1 Add 750 µl /slide of freshly prepared proteinase K solution on top of the section.
8.2 Incubate slides in a humidified chamber for 5 min at 38°C (HybEZ II oven).
8.3 Remove the solution by flicking the slide on absorbent paper.
8.4 Wash 2 times for 2 min in coplin jars filled with DEPC-treated water.

9. Protease III treatment for fixed -frozen sections (skip step for FFPE sections)

9.1 Change the HybEZ II oven temperature to 40°C.
9.2 Add 5-6 drops/slide of Protease III (from RNAScope H2O2 and Protease kit) on top of the section.
9.3 Incubate slides in a humidified chamber for 30 min at 40°C (HybEZ II oven).
9.4 Remove the solution by flicking the slide on absorbent paper.
9.5 Wash 2 times for 2 min in coplin jars filled with DEPC-treated water.

10. Hybridization

10.1 Change the HybEZ II oven temperature to 39°C.
10.2 Pre-warm hybridization buffer to 37°C.
10.3 Remove as much of the solution as possible from the slide with absorbent paper/kimwipe.
10.4 Add 250-300 µl of probe hybridization buffer on top of the tissue sample.
Note: Probe hybridization buffer contains formamide! Wear a mask suitable for organic solvents or work under a fume hood.
10.5 Pre-hybridize slides for 70-80 min in the humidified chamber at 39°C (HybEZ II oven).
10.6 Thaw probe stock mix (1 µM) on ice, vortex and spin down before using it.
10.7 Prepare probe solution by adding 0.6 µl - 4 µl of each probe mixture to a final volume of 80 µl/section probe hybridization buffer (**scale accordingly!**).
Note: The amount of probe stock used correspond to the abundance of the mRNA transcript & the number of probe oligos for detection of the target transcript.
10.8 Remove as much of the pre-hybridization solution as possible (with absorbent paper/kimwipe).
Note: Do this slide per slide.
10.9 Add 80 µl of probe solution on top of the tissue sample. Place a hybrislip on the slide (for equal distribution pf probe solution across the section).
10.10 Incubate **18-22 h** in the humidified chamber at 39°C (HybEZ II oven).

11. Prepare wash solutions (in coplin jars, cover the jars with parafilm to avoid evaporation and **pre-warm to 37°C** in incubator):

a. 100% probe wash buffer (40 ml)
b. 25% 5x SSCT (10 ml)/75% probe wash buffer (30 ml)
c. 50% 5x SSCT (20 ml)/50% probe wash buffer (20 ml)
d. 75% 5x SSCT (30 ml)/25% probe wash buffer (10 ml)
e. 100% 5x SSCT (40 ml)

Note: Probe wash buffer contains formamide! Wear a mask suitable for organic solvents or work under a fume hood.

12. Wash steps to remove excess probes

12.1 Remove the hybrislip without disturbing the tissue by immersing the slides in pre-warmed 100% probe wash buffer.
Note: Contains formamide! Dispose of the buffer accordingly!
12.2 Place the slides into coplin jar with pre-warmed 25% 5x SSCT/75% probe wash buffer for 15 min at 37°C (incubator)
12.3 Transfer slides into pre-warmed 50% 5x SSCT/50% probe wash buffer and incubate for 15 min at 37°C.
12.4 Transfer slides into pre-warmed 75% 5x SSCT/25% probe wash buffer and incubate for 15 min at 37°C.
12.5 Place the slides into the coplin jar with pre-warmed 5x SSCT for 15 min at 37°C.
12.6 Transfer the slides into coplin jar with fresh 5x SSCT and incubate 5 min at RT.

13. Pre-amplification

13.1 Move the amplification buffer to RT.
13.2 Replace blotting sheet in humidified chamber. Add DEPC-treated water to chamber if necessary.
13.3 Thaw hairpins **on ice** / in the **dark**!
13.4 Take off SSCT solution as much as possible with absorbent paper/kimwipe.
13.5 Add 400 µl/slide of amplification buffer on top of the tissue sample and pre-amplify in a humidified chamber for at least 60 min at RT.

14. Preparation of hairpin solution

14.1 Pipet separately 1.5 µl of each hairpin H1 and H2/per slide [3 µM stock in hairpin storage buffer] in separate PCR tubes.
14.2 In a PCR thermocycler: heat hairpins at 95 °C for 90 s.
14.3 Immediately put hairpins on ice for 5 minutes (in the dark).
14.4 Leave the hairpins at RT for 30 minutes IN THE DARK.
14.5 Prepare the hairpin solution by adding all snap-cooled hairpins to 75 µl of amplification buffer/slide at RT.

15. Amplification

15.1 Remove pre-amplification buffer as much as possible (one by one).
15.2 Add 75 µl of the hairpin solution on top of the tissue sample.
15.3 Place hybrislip on the solution.
15.4 Place the slide in humidified chamber.
15.5 Incubate overnight (**20-22h**) at RT and in the dark.

16. Remove hairpin (amplifier) excess

16.1 Immerse slides in 5x SSCT at RT to release hybrislip.
16.2 Remove excess hairpins by incubating the slides in coplin jars (covered with alufoil) at RT with 5x SSCT: **3 x 10 min**

17. Counterstain with DAPI

17.1 Move slides to a coplin jar with 40 ml 5xSSCT-DAPI 1:1000.
Note: Using water decreases signal intensity!
17.2 Incubate for 30 min in the dark.
17.3 Wash the slides for 5 min in 5xSSCT.

18. Mounting

18.1 Remove excess liquid with absorbent paper/kimwipes.
18.2 Immediately place 1-2 drops of ProLong Diamond Antifade Mountant on top of the section.
18.3 Carefully place a 22x40 mm glass coverslip over the tissue section. Avoid trapping air bubbles.
18.4 Dry slides overnight (in the dark).
18.5 Store slides at 4 °C protected from light.

Note: Samples are evaluated with a confocal microscope within 2 weeks; however, fluorescence was detectable even after 2 months of storage.

VII. LITERATURE CITED

Choi HMT, Schwarzkopf M, Fornace ME, et al. 2018. Third-generation *in situ* hybridization chain reaction: multiplexed, quantitative, sensitive, versatile, robust. *Development* 145:dev165753.

Meir J, Hartmann E, Eckstein M-T, et al. 2018. Identification of *Candida albicans* regulatory genes governing mucosal infection. *Cell Microbiol* 20:e12841.

Moreno-Velásquez SD, Pérez JC. 2021. Imaging and Quantification of mRNA Molecules at Single-Cell Resolution in the Human Fungal Pathogen *Candida albicans*. *mSphere* 6:e0041121.

VIII. RELATED READING AND RESOURCES

Molecular Instruments' HCR protocols
https://www.molecularinstruments.com/hcr-rnafish-protocols

ACDBio manual for RNAScope Multiplex Fluorescent Assay
https://acdbio.com/sites/default/files/UM%20323100%20Multiplex%20Fluorescent%20v2_RevB.pdf

A. Deryckere, R. Styfhals, A.M. Elagoz, E. Seuntjens. 2021. Hybridization Chain Reaction on paraffin sections. Available online (protocols.io):
https://protocols.io/view/hybridization-chain-reaction-on-paraffin-sectionsbxd7pi9n
