# Supplementary material for: An approach to analyze spatiotemporal patterns of gene expression at single-cell resolution in Candida albicans-infected mouse tongues
Source: mSphere. 2024 Aug 22;9(9):e00282-24. doi: 10.1128/msphere.00282-24 (PMC11423565; doi:10.1128/msphere.00282-24)
Supplement: Table S1 — Oligonucleotides used in the study. [file msphere.00282-24-s0003.pdf]

**Table S1. HCR split-initiator probes and corresponding HCR amplifier used in this study.**

| Name | Sequence (5´- 3´) | HCR Amplifier |
|---|---|---|
| CaRDN25_B3_1_A | gTCCCTgCCTCTATATCTTT**AAGTTCAGCGGGTAGTCCTACCTGA** | |
| CaRDN25_B3_1_B | **TCTTTTCCTCCGCTTATTGATATGC**TTCCACTCAACTTTAACCCg | |
| CaRDN25_B3_2_A | gTCCCTgCCTCTATATCTTT**CAACTCGGACGCCAAAGACGCCAGA** | |
| CaRDN25_B3_2_B | **GGGCCCAAAGATACCTTCTTCAAAT**TTCCACTCAACTTTAACCCg | |
| CaRDN25_B3_3_A | gTCCCTgCCTCTATATCTTT**CTGTTCCAAGGAACATAGACAAGAG** | |
| CaRDN25_B3_3_B | **CACGGGATTCTCACCCTCTGTGACG**TTCCACTCAACTTTAACCCg | |
| CaRDN25_B3_4_A | gTCCCTgCCTCTATATCTTT**ATGGAATTTACCACCCACTTAGAGC** | |
| CaRDN25_B3_4_B | **CGGTCTCTCGCCAATATTTAGCTTT**TTCCACTCAACTTTAACCCg | |
| CaRDN25_B3_5_A | gTCCCTgCCTCTATATCTTT**TGATCTCAAGCCCTTCCCTTTCAAC** | |
| CaRDN25_B3_5_B | **AGAGAGCAGCATGCAAAATACCAAG**TTCCACTCAACTTTAACCCg | B3 – Alexa Fluor 488 |
| CaRDN25_B3_6_A | gTCCCTgCCTCTATATCTTT**TGCTGGCCCGGTAAACCGCAGCGGC** | |
| CaRDN25_B3_6_B | **CGCCATTATCCTGCCGCTCCAAACC**TTCCACTCAACTTTAACCCg | |
| CaRDN25_B3_7_A | gTCCCTgCCTCTATATCTTT**CCTAGGTAAAAACCGCAGTCCTCGG** | |
| CaRDN25_B3_7_B | **GGCGACTTAAGATCATTATGCCAAC**TTCCACTCAACTTTAACCCg | |
| CaRDN25_B3_8_A | gTCCCTgCCTCTATATCTTT**GTACGACTTGGCATGAAAACTATTC** | |
| CaRDN25_B3_8_B | **AACCTTGGAGACCTGCTGCGGTTAT**TTCCACTCAACTTTAACCCg | |
| CaRDN25_B3_9_A | gTCCCTgCCTCTATATCTTT**GCTAGAACGTGGAGTTTGACCTCCA** | |
| CaRDN25_B3_9_B | **CAACCCGGATCGCCCAGAGGGCTTA**TTCCACTCAACTTTAACCCg | |
| CaRDN25_B3_10_A | gTCCCTgCCTCTATATCTTT**AGCGCACCGTTGCCAGCCTGCTATG** | |
| CaRDN25_B3_10_B | **CGGCAAGCGCACAAAGCCTTTCCGC**TTCCACTCAACTTTAACCCg | |
| CaRDN25_B2_1_A | CCTCgTAAATCCTCATCAAA**AAGTTCAGCGGGTAGTCCTACCTGA** | |
| CaRDN25_B2_1_B | **TCTTTTCCTCCGCTTATTGATATGC**AAATCATCCAgTAAACCgCC | |
| CaRDN25_B2_2_A | CCTCgTAAATCCTCATCAAA**CAACTCGGACGCCAAAGACGCCAGA** | |
| CaRDN25_B2_2_B | **GGGCCCAAAGATACCTTCTTCAAAT**AAATCATCCAgTAAACCgCC | |
| CaRDN25_B2_3_A | CCTCgTAAATCCTCATCAAAC**TGTTCCAAGGAACATAGACAAGAG** | |
| CaRDN25_B2_3_B | **CACGGGATTCTCACCCTCTGTGACG**AAATCATCCAgTAAACCgCC | |
| CaRDN25_B2_4_A | CCTCgTAAATCCTCATCAAA**ATGGAATTTACCACCCACTTAGAGC** | |
| CaRDN25_B2_4_B | **CGGTCTCTCGCCAATATTTAGCTTT**AAATCATCCAgTAAACCgCC | |
| CaRDN25_B2_5_A | CCTCgTAAATCCTCATCAAA**TGATCTCAAGCCCTTCCCTTTCAAC** | |
| CaRDN25_B2_5_B | **AGAGAGCAGCATGCAAAATACCAAG**AAATCATCCAgTAAACCgCC | B2 – Alexa Fluor 647 |
| CaRDN25_B2_6_A | CCTCgTAAATCCTCATCAAA**TGCTGGCCCGGTAAACCGCAGCGGC** | |
| CaRDN25_B2_6_B | **CGCCATTATCCTGCCGCTCCAAACC**AAATCATCCAgTAAACCgCC | |
| CaRDN25_B2_7_A | CCTCgTAAATCCTCATCAAA**CCTAGGTAAAAACCGCAGTCCTCGG** | |
| CaRDN25_B2_7_B | **GGCGACTTAAGATCATTATGCCAAC**AAATCATCCAgTAAACCgCC | |
| CaRDN25_B2_8_A | CCTCgTAAATCCTCATCAAA**GTACGACTTGGCATGAAAACTATTC** | |
| CaRDN25_B2_8_B | **AACCTTGGAGACCTGCTGCGGTTAT**AAATCATCCAgTAAACCgCC | |
| CaRDN25_B2_9_A | CCTCgTAAATCCTCATCAAA**GCTAGAACGTGGAGTTTGACCTCCA** | |
| CaRDN25_B2_9_B | **CAACCCGGATCGCCCAGAGGGCTTA**AAATCATCCAgTAAACCgCC | |
| CaRDN25_B2_10_A | CCTCgTAAATCCTCATCAAA**AGCGCACCGTTGCCAGCCTGCTATG** | |
| CaRDN25_B2_10_B | **CGGCAAGCGCACAAAGCCTTTCCGC**AAATCATCCAgTAAACCgCC | |
| CaHWP1_B2_1_A | CCTCgTAAATCCTCATCAAA**TAAGCGATAGCAATAAGTTGAGCAG** | |
| CaHWP1_B2_1_B | **ACAGTGGCCCCAATTGATAACATGT**AAATCATCCAgTAAACCgCC | |
| CaHWP1_B2_2_A | CCTCgTAAATCCTCATCAAA**GTTGTTGTGGGTAATCACAAGGCTC** | |
| CaHWP1_B2_2_B | **GGTAATCACAAGGTTCTTCCTGCTG**AAATCATCCAgTAAACCgCC | |
| CaHWP1_B2_3_A | CCTCgTAAATCCTCATCAAA**GCTGTTGTGGATAGTCACATGGCTC** | |
| CaHWP1_B2_3_B | **GTTGTGGGTAGTCACAAGGTTCTTG**AAATCATCCAgTAAACCgCC | |
| CaHWP1_B2_4_A | CCTCgTAAATCCTCATCAAA**GTGGGTAGTCACAAGGTTCTTGTGG** | |
| CaHWP1_B2_4_B | **GATTGTCGCAAGGTTCTTGTGGTTG**AAATCATCCAgTAAACCgCC | |
| CaHWP1_B2_5_A | CCTCgTAAATCCTCATCAAA**GAGGAGGATTGTCACAAGGAACATC** | |
| CaHWP1_B2_5_B | **GAGGATTGTCACAAGGAACATCAGG**AAATCATCCAgTAAACCgCC | B2 – Alexa Fluor 647 |
| CaHWP1_B2_6_A | CCTCgTAAATCCTCATCAAA**GATTGTCACAAGGAACATCAGGTTG** | |
| CaHWP1_B2_6_B | **TGTCACAAGGAACATCAGGTTGAGG**AAATCATCCAgTAAACCgCC | |
| CaHWP1_B2_7_A | CCTCgTAAATCCTCATCAAA**GTCATCAGGCTGATCAGGTTGAGGA** | |
| CaHWP1_B2_7_B | **GGTTGGAATGTTTGGAATAGGAGGA**AAATCATCCAgTAAACCgCC | |
| CaHWP1_B2_8_A | CCTCgTAAATCCTCATCAAA**AACCAGCAGGAATTGTTTCCATAGG** | |
| CaHWP1_B2_8_B | **TTTCACCGGCAGGCATGGATGGTTC**AAATCATCCAgTAAACCgCC | |
| CaHWP1_B2_9_A | CCTCgTAAATCCTCATCAAA**AGCTGATTCAGTAGCTGGAACATCT** | |
| CaHWP1_B2_9_B | **ACCAGCTGGAGTCATTTCAGGAACT**AAATCATCCAgTAAACCgCC | |
| CaHWP1_B2_10_A | CCTCgTAAATCCTCATCAAA**TTTCACCGGCAGGAATAGATGGTTG** | |
| CaHWP1_B2_10_B | **CATCTGATTTTGGAACAGCTGGAGA**AAATCATCCAgTAAACCgCC | |
| CaECE1_B3_1_A | gTCCCTgCCTCTATATCTTT**CATGTTGAATTCTGGAGCATGGTGG** | |
| CaECE1_B3_1_B | **TGGGGCAGCTGGAGCAACATCTCTC**TTCCACTCAACTTTAACCCg | |
| CaECE1_B3_2_A | gTCCCTgCCTCTATATCTTT**CAGTAGGTGCTTGGTCAGCTGGAGC** | |
| CaECE1_B3_2_B | **CAGTATTGAATTCTTGAGGTGCAGG**TTCCACTCAACTTTAACCCg | |
| CaECE1_B3_3_A | gTCCCTgCCTCTATATCTTT**TGGTAGAAGCAACAGAAGTCATGGC** | |
| CaECE1_B3_3_B | **CAACGTCATCATTAGCTCCATCTCT**TTCCACTCAACTTTAACCCg | |
| CaECE1_B3_4_A | gTCCCTgCCTCTATATCTTT**CAATTTCTGGCAATCTGACGACGGC** | |
| CaECE1_B3_4_B | **GTTGAACACCAGTGGCAACACGAGC**TTCCACTCAACTTTAACCCg | B3 – Alexa Fluor 488 |
| CaECE1_B3_5_A | gTCCCTgCCTCTATATCTTT**ACGCCATCTCTCTTGGCATTTTCGA** | |
| CaECE1_B3_5_B | **GCAACAAGATTAAGGCCAACATCTG**TTCCACTCAACTTTAACCCg | |

| | | |
|---|---|---|
| CaECE1_B3_6_A | gTCCCTgCCTCTATATCTTT**TCCAGGACGCCATCAAAAACGTTAG** | |
| CaECE1_B3_6_B | **CCATCTCTCTTAGCTTGTTGAACAG**TTCCACTCAACTTTAACCCg | |
| CaECE1_B3_7_A | gTCCCTgCCTCTATATCTTT**GTGGGAGTCTTTGAAGAAGTTCATC** | |
| CaECE1_B3_7_B | **CAGATTCAGCTGATCTAGTAATGAG**TTCCACTCAACTTTAACCCg | |
| CaECE1_B3_8_A | gTCCCTgCCTCTATATCTTT**CATCTCTTTTAACTGGTTGACTGTC** | |
| CaECE1_B3_8_B | **TTAAATTGCTAAGTGCTACTGAGCC**TTCCACTCAACTTTAACCCg | |
| CaZRT1_B2_1_A | CCTCgTAAATCCTCATCAAA**AGCTCCGTGGTTGCTAATCTGTAGC** | |
| CaZRT1_B2_1_B | **AAGTAAACAGTACCATCAGTAGGGG**AAATCATCCAgTAAACCgCC | |
| CaZRT1_B2_2_A | CCTCgTAAATCCTCATCAAA**AACACTAGCAGACAGGTTCAGTGGT** | |
| CaZRT1_B2_2_B | **GTGACAGTCAGTAACAGCTGTTGTT**AAATCATCCAgTAAACCgCC | |
| CaZRT1_B2_3_A | CCTCgTAAATCCTCATCAAA**CCATCAACACAATATTGGACCGAAT** | |
| CaZRT1_B2_3_B | **GGCAAGATTGAACCTTCATTTCCGT**AAATCATCCAgTAAACCgCC | |
| CaZRT1_B2_4_A | CCTCgTAAATCCTCATCAAA**GCTTGTAGGAAGGTTGTTTGTATTG** | |
| CaZRT1_B2_4_B | **ACCATCGTGGGAATGGCATCCATCA**AAATCATCCAgTAAACCgCC | |
| CaZRT1_B2_5_A | CCTCgTAAATCCTCATCAAA**TGCTCAACACCAGCATGGAAATGAC** | |
| CaZRT1_B2_5_B | **GCGTCATGGTTATTGTCATCGACA**CAAATCATCCAgTAAACCgCC | B2 – Alexa Fluor 647 |
| CaZRT1_B2_6_A | CCTCgTAAATCCTCATCAAA**CAAAAAGCAACCCAATTCTCAAAGG** | |
| CaZRT1_B2_6_B | **ACCCGATACCAGAAGTAACAAGAAT**AAATCATCCAgTAAACCgCC | |
| CaZRT1_B2_7_A | CCTCgTAAATCCTCATCAAA**GCCGTTGATATGATTATACCAGTAC** | |
| CaZRT1_B2_7_B | **TGCGCATGAGTCATTAAATGGACAA**AAATCATCCAgTAAACCgCC | |
| CaZRT1_B2_8_A | CCTCgTAAATCCTCATCAAA**GAAGCACCAGTACCTTCATATTTGA** | |
| CaZRT1_B2_8_B | **GCAATAAAAATCCCAGCCATGGTTA**AAATCATCCAgTAAACCgCC | |
| CaZRT1_B2_9_A | CCTCgTAAATCCTCATCAAA**GAGTGCCATCACTAATTTAGTTGAC** | |
| CaZRT1_B2_9_B | **TCCAATAGGGGTGATCAATGCAAAC**AAATCATCCAgTAAACCgCC | |
| CaZRT1_B2_10_A | CCTCgTAAATCCTCATCAAA**TTCAATTAAACCAGTCCAAAGGAGC** | |
| CaZRT1_B2_10_B | **GCCGTGTAACCAATCGTGACTCCAC**AAATCATCCAgTAAACCgCC | |
| CaPRA1_B1_1_A | gAggAgggCAgCAAACggAA**GTAACCGTAACTGGTGCAGCAACGG** | |
| CaPRA1_B1_1_B | **CCTGTAGGTGAAGCATCAACAAATC**TAgAAgAgTCTTCCTTTACg | |
| CaPRA1_B1_2_A | gAggAgggCAgCAAACggAA**AAACCTTTAACCCAGTCGGCCCGCC** | |
| CaPRA1_B1_2_B | **GTGGCATTACACGACAGATCAATCG**TAgAAgAgTCTTCCTTTACg | |
| CaPRA1_B1_3_A | gAggAgggCAgCAAACggAA**AATAATTGAGCTTCTTGCAACCCAG** | |
| CaPRA1_B1_3_B | **AATGTGTGGTCCCTGGCATGTTCAG**TAgAAgAgTCTTCCTTTACg | |
| CaPRA1_B1_4_A | gAggAgggCAgCAAACggAA**ATGACCAACAACCTCAGCACTTGCA** | |
| CaPRA1_B1_4_B | **TTTGTCAGCACCGACAACATTGTCA**TAgAAgAgTCTTCCTTTACg | |
| CaPRA1_B1_5_A | gAggAgggCAgCAAACggAA**ACCAGCCCAGCCATCATTTTTGCAC** | |
| CaPRA1_B1_5_B | **ATCGCTATGGTTGGAACCTCTCCAA**TAgAAgAgTCTTCCTTTACg | B1 – Alexa Fluor 546 |
| CaPRA1_B1_6_A | gAggAgggCAgCAAACggAA**GGAGCATAGTTGGGTTAAGTATCTT** | |
| CaPRA1_B1_6_B | **CTTAGATTTCGAGACGGTATATCCA**TAgAAgAgTCTTCCTTTACg | |
| CaPRA1_B1_7_A | gAggAgggCAgCAAACggAA**GTGTAACAAGTCACCTGCCCAAAAA** | |
| CaPRA1_B1_7_B | **GACCAATCGATTTCAAGTGCCAGAA**TAgAAgAgTCTTCCTTTACg | |
| CaPRA1_B1_8_A | gAggAgggCAgCAAACggAA**GTCACATCATATGCATACACATCCA** | |
| CaPRA1_B1_8_B | **TCTCCATTGCACCCTTCGCCGGG**AATAgAAgAgTCTTCCTTTACg | |
| CaPRA1_B1_9_A | gAggAgggCAgCAAACggAA**CGCTATCCTCGAAGCTGCTAAAATC** | |
| CaPRA1_B1_9_B | **TACTGGCCCCTGAATCAGAGCCACT**TAgAAgAgTCTTCCTTTACg | |
| CaPRA1_B1_10_A | gAggAgggCAgCAAACggAA**GTATGTTGATGAGAACTTGAGGCTG** | |
| CaPRA1_B1_10_B | **TCTGTTGTGGCGCTAGGGTTGCTAT**TAgAAgAgTCTTCCTTTACg | |
| MmKRT13_B1_1_A | gAggAgggCAgCAAACggAA**AAGCGGCAGCTCATGGTGGGAGCAA** | |
| MmKRT13_B1_1_B | **CCTCCATAGCTCATGGAGGAACTCT**TAgAAgAgTCTTCCTTTACg | |
| MmKRT13_B1_2_A | gAggAgggCAgCAAACggAA**TCCAAGCTGGCAAGAACCAGCTCCG** | |
| MmKRT13_B1_2_B | **TGAGCAGGAGGAGATATTACGGCCT**TAgAAgAgTCTTCCTTTACg | |
| MmKRT13_B1_3_A | gAggAgggCAgCAAACggAA**TCCAGCTGACCCTCCAGTGACAAAC** | |
| MmKRT13_B1_3_B | **GCCACAGCTCATGCCCCCTCCATAG**TAgAAgAgTCTTCCTTTACg | |
| MmKRT13_B1_4_A | gAggAgggCAgCAAACggAA**AAAGCCACCGCCAAAACCTCCGCCA** | |
| MmKRT13_B1_4_B | **GCCACCATCGACACCTCCGAAGTCA**TAgAAgAgTCTTCCTTTACg | |
| MmKRT13_B1_5_A | gAggAgggCAgCAAACggAA**TGCCTCCAAGGCGCGCACCTTATCC** | |
| MmKRT13_B1_5_B | **AATCTTCACCTCCAGGTCGGCATTG**TAgAAgAgTCTTCCTTTACg | B1 – Alexa Fluor 546 |
| MmKRT13_B1_6_A | gAggAgggCAgCAAACggAA**ATCTCCACATTGACCTGGCCTACTA** | |
| MmKRT13_B1_6_B | **GTCAGATCAATGCCAGGGGTGGCGT**TAgAAgAgTCTTCCTTTACg | |
| MmKRT13_B1_7_A | gAggAgggCAgCAAACggAA**CATCCCTCCGATTCTTCTCTGCCAG** | |
| MmKRT13_B1_7_B | **CACTCTTGGTCTGGAACCATTCCTC**TAgAAgAgTCTTCCTTTACg | |
| MmKRT13_B1_8_A | gAggAgggCAgCAAACggAA**TGGAGGATACTTCCTTGTTCAGCTC** | |
| MmKRT13_B1_8_B | **TCTTGCTGGTCTGGATCATTTCAGC**TAgAAgAgTCTTCCTTTACg | |
| MmKRT13_B1_9_A | gAggAgggCAgCAAACggAA**AGCTCGCTCAGCTGAGCCTCGATGC** | |
| MmKRT13_B1_9_B | **TGGTTCTGGCACTCCATCTCACTTC**TAgAAgAgTCTTCCTTTACg | |
| MmKRT13_B1_10_A | gAggAgggCAgCAAACggAA**AGGAGGGGGACCCGTTGGAGGTAGT** | |
| MmKRT13_B1_10_B | **TTCGGAAATCTGGGCGACCGGAATT**TAgAAgAgTCTTCCTTTACg | |
